# Observing non-Hermiticity induced chirality breaking in a synthetic Hall ladder

**DOI:** 10.1038/s41377-024-01700-1

**Published:** 2025-01-08

**Authors:** Rui Ye, Yanyan He, Guangzhen Li, Luojia Wang, Xiaoxiong Wu, Xin Qiao, Yuanlin Zheng, Liang Jin, Da-Wei Wang, Luqi Yuan, Xianfeng Chen

**Affiliations:** 1 State Key Laboratory of Advanced Optical Communication Systems and Networks, School of Physics and Astronomy, Shanghai Jiao Tong University, Shanghai, 200240 China; 2 School of Physics, Nankai University, Tianjin, 300071 China; 3 Zhejiang Key Laboratory of Micro-Nano Quantum Chips and Quantum Control, School of Physics, and State Key Laboratory for Extreme Photonics and Instrumentation, Zhejiang University, Hangzhou, 310027 China; 4 Shanghai Research Center for Quantum Sciences, Shanghai, 201315 China; 5 Collaborative Innovation Center of Light Manipulations and Applications, Shandong Normal University, Jinan, 250358 China

**Keywords:** Optics and photonics, Optical physics

## Abstract

Non-Hermitian topological photonics plays a key role in bridging topological matter with gain and loss engineering in optics. Here we report the experimental observation of the break of chiral currents in a Hall ladder from the non-Hermiticity by constructing synthetic frequency dimension in two rings, where currents on both legs of the ladder co-propagate in the same direction. The origin of such phenomena is resulted from the interplay between the effective magnetic flux and the on-site gain and loss. Such non-Hermitian co-propagating currents exhibit characteristics of unidirectional frequency conversion in both rings, and moreover, different from the counterpart in Hermitian systems, can provide a method to probe the signatures of the non-Hermitian skin effect from steady-state bulk dynamics. Our model is further extended to models including next-nearest-neighbor couplings, pointing to a way for observing the non-Hermitian signature with higher winding number, and provides a new control knob for light manipulation with the topological dissipation engineering.

## Abstract (web summary)

The non-Hermiticity of gain/loss can break the chiral currents in a Hall ladder, resulting in antichiral currents where currents on both legs co-propagate in the same direction.

## Graphical abstract

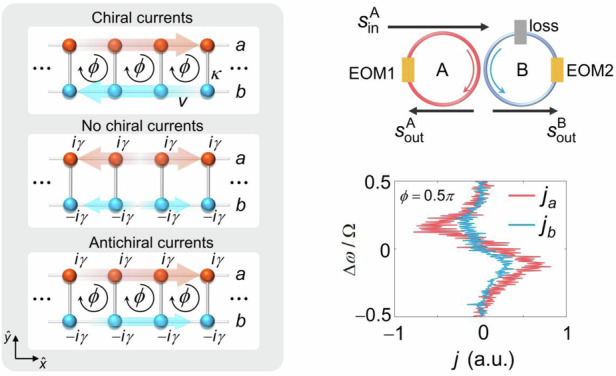

## Introduction

Chiral currents at opposite boundaries of two-dimensional (2D) topological materials are featured by robust one-way transport but in opposite directions^1–7^. Recently, it was found that the broken chirality in a topological system leads to anomalous topological phenomena with the so-called antichiral currents^8–17^, where edge currents at two boundaries co-propagate in the same direction. This counterintuitive phenomenon complements chiral currents, bringing a new control knob to functional wave guiding^18^. However, such chiral symmetry breaking in edge currents has only been experimentally demonstrated in Hermitian microwave systems^19–22^, which holds difficulty in extending to the optical frequency regime.

On the other hand, it has been recently noticed that the construction of photonic models with the synthetic dimensions may provide a versatile way in studying many topological and non-Hermitian physics due to its unique capacity for introducing effective gauge potentials and performing gain-loss engineering^23–25^. Besides using degrees of freedom of light such as modes^7^, time^26^, and orbital angular momentum^27^ to form synthetic dimensions, the construction of the discrete lattice Hamiltonian using the frequency axis of light^28–35^ has been successful in studying various physics including chiral currents^36^ in the quantum Hall ladder and measuring topological windings^37^. In these experiments, fiber-based rings under dynamic modulation have been used to simulate different physical phenomena, with great flexibility in light manipulation and potential scalability to the on-chip applications. It is therefore of fundamental curiosity to seek the realization of antichiral currents using synthetic frequency dimension.

In this work, we unveil a different physical origin of broken chirality^38^ in the non-Hermitian platform and report the experimental demonstration in the optical regime. This is achieved by using synthetic frequency dimension in two rings, where a non-Hermitian Hall ladder is constructed with different losses and hopping phases on the two legs. The advantages in controlling the light from non-Hermiticity^39–48^ and the effective gauge potential for photons^4,36^ are combined, so currents on both legs co-propagate in the same direction and then exhibit unidirectional frequency conversion in both rings. Fundamentally different from microwave Hermitian antichiral currents^18–20^, the observed non-Hermitian co-propagating currents can exhibit intrinsic signatures of the non-Hermitian skin effect^38,49–66^ from steady-state bulk dynamics without obtaining the Lyapunov exponent^67,68^, which is verified by measuring nontrivial topological windings of band structures in the complex energy plane in our experiments^69–73^. The universality of this strategy is verified by extending the model to the Hall ladder lattice including next-nearest-neighbor (NNN) couplings. Our results hence realize optical antichiral currents from non-Hermitian topology in a two-leg ladder model, demonstrating the unidirectional frequency conversion in the telecommunications band and providing a protocol that can be generalized to other electromagnetic wavelengths.

## Results

### Theoretical analysis

We study a non-Hermitian two-leg Hall ladder model described by the Hamiltonian

1 $$H=\sum _{n}i\gamma \left({a}_{n}^{\dagger }{a}_{n}-{b}_{n}^{\dagger }{b}_{n}\right)+\kappa \sum _{n}\left({a}_{n}^{\dagger }{b}_{n}+{b}_{n}^{\dagger }{a}_{n}\right)+\sum _{n}\left[\left(v{e}^{-i\phi /2}{a}_{n}^{\dagger }{a}_{n+1}+v{e}^{i\phi /2}{b}_{n}^{\dagger }{b}_{n+1}\right)+h.c.\right]$$

where ${a}_{n}^{\dagger }({b}_{n}^{\dagger })$ and ${a}_{n}({b}_{n})$ are creating and annihilation operators of $n$-th lattice site in legs $a$ and $b$, respectively. $\kappa$ is the coupling strength between two sites on each leg, and $v$ describes the nearest-neighbor hopping strength between two sites on each leg. The difference between hopping phases on two legs gives the effective magnetic flux $\phi$ for photons. There are on-site gain (*iγ*) and loss (*-iγ*) on the leg $a$ and $b$, respectively. If the lattice is infinite, the corresponding Bloch Hamiltonian in the momentum space is

2 $${H}_{k}=\left[\begin{array}{cc}i\gamma +2v\,\cos (k-\frac{\phi }{2}) & \kappa \\ \kappa & -i\gamma +2v\,\cos (k+\frac{\phi }{2})\end{array}\right]$$

where $k\in \left(0,2\pi \right]$ is the Bloch wave number. The corresponding band structure is

3 $${E}_{1,2}(k)=2v\,\cos k\,\cos \frac{\phi }{2}\pm \sqrt{{\left(2v\sin k\sin \frac{\phi }{2}+i\gamma \right)}^{2}+{\kappa }^{2}}$$

and corresponding eigenstates ${\psi }_{1,2}=\left({\psi }_{1,2}^{a},{\psi }_{1,2}^{b}\right)$ with ${\psi }_{1,2}^{a}$ and ${\psi }_{1,2}^{b}$ being the components on the legs $a$ and $b$, respectively.

**Fig. 1:**
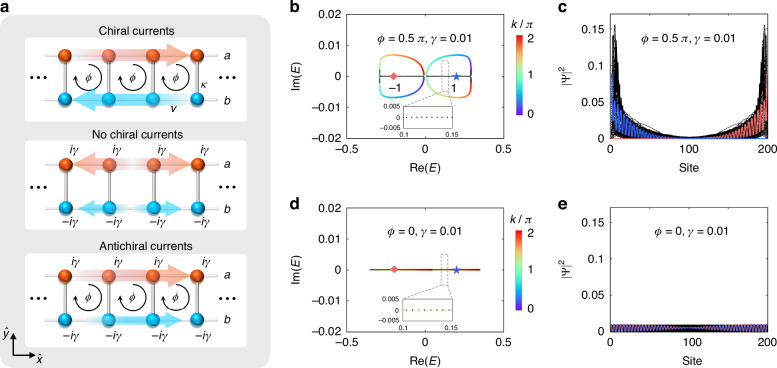
**Chiral currents and antichiral currents with corresponding non-Hermitian skin effect. a** The comparison of chiral currents, no chiral currents, and antichiral currents in a two-leg ladder model. The red and blue arrows denote the currents on the leg $a$ and $b$, respectively. **b**, **d** Energy spectra under the periodic boundary condition (colored lines) and open boundary condition (black dots) for lattices with $\phi =0.5\pi$ (**b**) and $\phi =0$ (**d**), wherein the red diamonds and blue stars denote the eigenenergies with $\text{Re}(E)=\pm 0.2$. The numbers in the figures indicate the value of $w$ in different loops. The inserted figures show the zoom-in energy spectra in the dashed boxes. **c**, **e** All the eigenstates of the lattices under the open boundary condition with $\phi =0.5\pi$ (**c**) and $\phi =0$ (**e**). The red and blue lines highlight the eigenstates of $\text{Re}(E)=\pm 0.2$, i.e., the red diamonds and blue stars in (**b**) and (**d**). Other parameters are $\kappa =0.15,v=0.1$, and $\gamma =0.01$

When the on-site gain/loss are absent $(\gamma =0)$, the two-leg Hall ladder is Hermitian and can exist chiral currents, i.e., the edge states on either leg ($a$ or $b$) propagate in opposite directions, for the nonzero magnetic flux ($\phi \ne 0$ or $\pi$) (see the top panel in Fig. 1a), which manifests the chiral edge states of a 2D quantum Hall insulator even if the entire bulk lattices are removed^5,36,74^. For the case of zero magnetic flux $(\phi =0)$, the chiral currents are absent (see the middle panel in Fig. 1a) no matter if there exists on-site gain/loss. However, when the magnetic flux and on-site gain/loss both exist ($\phi\,\ne\, 0$ or $\pi$, $\gamma\,\ne\, 0\left)\right.$, the currents on two legs co-propagate in the same direction (see the down panel in Fig. 1a), resulting in the so-called antichiral currents in this model. It has also been noted that such antichiral currents can drive the bulk eigenstates to localize near the boundaries of the lattice under open boundary conditions, which leads to the non-Hermitian skin effect associated to the phenomena of the energy localization of all bulk modes on the lattice boundary^70^. We can characterize the skin effect by the winding number, originating from the point-gap topology of band structures^71^

4 $$w=\mathop{\sum}\limits_{i=1,2}\mathop{\int}\nolimits_{0}^{2\pi}{\frac{dk}{2\pi}}{\partial}_{k}{\rm{arg}}[{E}_{i}(k)-\varepsilon ]$$

where $\varepsilon$ is a reference energy in the complex energy plane. The nonzero winding number ($w\ne 0$) indicates the existence of the skin effect, and the sign of $w$ determines the direction of the skin effect^69–73^. The skin effect is absent when the winding number is zero ($w=0$). In Fig. 1b, d, we present the corresponding energy spectra with the periodic boundary condition and open boundary condition in the complex energy plane for different effective magnetic flux $\phi$ and nonzero gain/loss ($\gamma =0.01$), where the values of $w$ are given inside each loop. Distributions of all the eigenstates are shown in Fig. 1c, e, where two eigenstates of $\mathrm{Re}(E)=\pm 0.2$ (blue stars and red diamonds in Fig. 1b, d) are highlighted in blue and red lines. The energy spectra under the periodic boundary condition form two closed loops, one of which has $w=1(-1)$ for ${\text{Re}}(E)\,>\, 0[{\text{Re}}(E)\,<\, 0]$, indicating the corresponding eigenstates $\left(\Psi \right)$ localized on the left (right) side of the lattice for the nonzero magnetic flux of $\phi =0.5\pi$ (see Fig. 1c). This is the so-called bipolar non-Hermitian skin effect^55,56^, where eigenstates can localize on both sides of the lattice depending on the eigenenergies and has only been demonstrated in acoustic systems^56^. However, the energy spectra under both boundary conditions overlap for zero effective magnetic flux ($\phi =0$), and thus the winding number is zero ($w=0$), indicating the absence of the skin effect (see Fig. 1d, e). Thus, the skin effect can be controlled by the effective magnetic flux in the non-Hermitian two-leg Hall ladder.

**Fig. 2:**
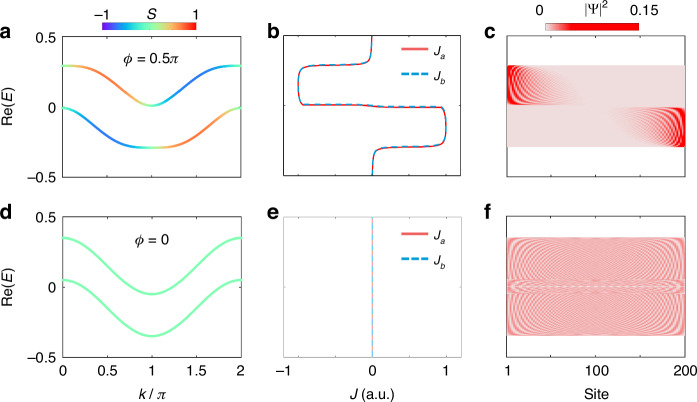
**Correspondence between antichiral currents and the non-Hermitian skin effect. a**, **d** Theoretical band structures. The color shows the asymmetry ratio $S$. **b**, **e** Calculated currents ${J}_{a}$ and ${J}_{b}$ for different eigenenergies. **c**, **f** Distributions of all the eigenstates under the open boundary condition. **a**–**c** With effective magnetic flux ($\phi =0.5\pi$). **d**–**f** Without effective magnetic flux ($\phi =0$). Other parameters are the same as those used in Fig. 1

In such a model, the underlying skin effect originates from the interplay between the effective magnetic flux and on-site gain/loss. In Fig. 2a, d, we plot band structures for effective magnetic flux $\phi =0.5\pi$ and $\phi =0$ with the asymmetry ratio $S$, defined as $S=({|{\psi}^{a}|}^{2}-{|{\psi }^{b}|}^{2})/({|{\psi }^{a}|}^{2}+{|{\psi }^{b}|}^{2})$. $S=1(-1)$ means the eigenstates mainly locate on the leg $a(b)$. When $\phi =0.5\pi$, we can see the eigenstates on the two legs $a$ and $b$ have the opposite dispersion for a specific eigenenergy, which are the chiral currents as signatures from the Hermitian topology. For example, for the eigenenergy with $\text{Re}(E)=-0.2$, the current on the leg $a(b)$ propagates along $+\hat{x}(-\hat{x})$ axis, due to the positive (negative) dispersion. However, the phenomenon of the chiral currents may disappear once the on-site gain/loss is added. On the leg $a$ with gain (*iγ*), the current along $+\hat{x}$ axis does not change its direction, as it experiences gain and gets increased. However, the original current on the leg $b$ along the $-\hat{x}$ axis gradually decays to zero due to the loss; meanwhile, the increasing current on the leg $a$ leaks into the leg $b$ via the coupling between two legs. As a result, the interplay between these two trends changes the current direction on the leg $b$ and makes it co-propagate along $+\hat{x}$ direction, which are denoted as the antichiral currents in this two-leg model.

We can quantitively describe the antichiral currents using the current definition on each leg^74^

5 $${J}_{a(b)}(E)=\sum _{i=1,2}\int dk\delta ({E}_{i}-E)|\langle {\psi }_{i}|a(b)\rangle {|}^{2}\frac{\partial {\rm{Re}}[{E}_{i}(k)]}{\partial k}$$

where $\partial \text{Re}[{E}_{i}(k)]/\partial k$ represents the group velocity, and $\delta ({E}_{i}-E)$ is the Dirac delta function which characterizes the density of states. Considering the lifetime of the states, we can express the density of states as

6 $$\int dk\delta ({E}_{i}-E)=\sum _{k}\frac{1}{\pi }\frac{{\rm{Im}}[{E}_{i}(k)]}{{\{{E}_{i}(k)-{\rm{Re}}[{E}_{i}(k)]\}}^{2}+{\{{\rm{Im}}[{E}_{i}(k)]\}}^{2}}$$

where $\text{Im}[{E}_{i}(k)]$ is the imaginary part of the eigenenergy which indicates the lifetime of the eigenstates. From Eqs. (5) and (6), we see the sign of the current on each leg [${J}_{a(b)}]$ is determined by the group velocity $\partial \text{Re}\left[{E}_{i}\left(k\right)\right]/\partial k$, projection of eigenstates on the leg $a(b)$, ${|{\rm{\langle }}{\psi }_{i}{|a}(b){\rm{\rangle }}|}^{2}$, and the sign of $\text{Im}[{E}_{i}(k)]$. If ${J}_{a(b)}(E)\,>\, 0$, the current on the leg $a(b)$ propagates along $+\hat{x}$ direction. In contrast, if ${J}_{a(b)}(E)\,<\, 0$, the current propagates along $-\hat{x}$ direction. The antichiral currents can be demonstrated if the currents on each leg propagate in the same direction, i.e., ${J}_{a}(E),{J}_{b}(E)\,>\,0(\,<\, 0)$.

To confirm the existence of antichiral currents and the correspondence between the antichiral currents and the skin effect, we further plot distributions of the eigenstates for all eigenenergies under the open boundary condition for different effective magnetic flux $\phi$ and nonzero gain/loss ($\gamma =0.01$). The correspondence between the currents ${J}_{a},{J}_{b}$ (antichiral currents) and the eigenstate distributions can be noticed by comparing Fig. 2b, e and Fig. 2c, f. When the magnetic flux is $0.5\pi$, one can see ${J}_{a}(E)={J}_{b}(E)\,<\,0(\,>\,0)$ for ${\rm{Re}}(E)\,>\,0[{\rm{Re}}(E) \,<\, 0]$, thus showing the direction of antichiral currents is along $-\hat{x}(+\hat{x})$ for the upper (lower) band (see Fig. 2b). The antichiral currents are consistent with the skin effect, i.e., the eigenstates localize at the left (right) boundary of the lattice for ${J}_{a},{J}_{b} \,<\, 0({J}_{a},{J}_{b} \,>\, 0)$ (see Fig. 2c). The boundary of the localized states agrees well with the direction of ${J}_{a(b)}$ for each eigenenergy. The antichiral currents emerge once the gain/loss is added (details are in Supplementary Note 1). We note that the boundary where the distribution of the eigenstate is localized is dependent on the real value of eigenenergy $E$, i.e., the localization is at left (right) for ${\rm{Re}}\,(E)\,>\, 0[{\rm{Re}}\,(E)\,<\, 0]$. Such phenomena correspond to the bipolar non-Hermitian skin effect that we discussed in Fig. 1c. Moreover, for zero magnetic flux $(\phi =0)$, we notice there is no existence of antichiral currents $[{J}_{a}(E)={J}_{b}(E)=0]$ (see Fig. 2e). The skin effect also disappears in this case, as shown in Fig. 2f. The cases of other phases are discussed in Supplementary Note 2.

### Experimental demonstrations

**Fig. 3:**
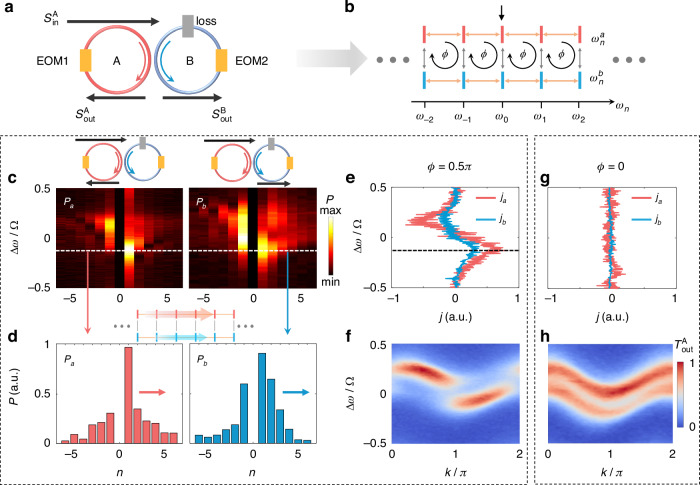
**Experimental configuration and measurement of the antichiral currents. a** Two identical coupled fiber ring resonators (A, B) are modulated by two electro-optic phase modulators (EOM1, EOM2). The black arrows denote the input and drop-port fibers for inject and readout light. **b** The synthetic two-leg Hall ladder in the frequency space of the two coupled ring resonators. The black arrow represents the initial excitation of the input laser. **c** Experimentally measured steady-state normalized mode distributions ${P}_{a}(n)$ and ${P}_{b}(n)$ for $\phi =0.5\pi$. The strong signal on the excited mode $n=0$ has been removed. The top panels denote that the ${P}_{a}$ and ${P}_{b}$ are measured from the drop-port output fields of ring A and B, respectively. **d** Measured steady-state normalized mode distributions ${P}_{a}(n)$ and ${P}_{b}(n)$ for the frequency detuning of $\Delta \omega =-0.12\Omega$ with $\phi =0.5\pi$, i.e., the white dashed lines in (**c**). The red and blue arrows in the figure denote the directions of currents on each leg. The top panel denotes the schematic of positive antichiral currents for this frequency detuning. **e** Measured steady-state chiral currents ${j}_{a},{j}_{b}$ for $\phi =0.5\pi$. **f** Measured projected band structures for $\phi =0.5\pi$. **g,** **h** Measured ${j}_{a},{j}_{b}$ and projected band structures for $\phi =0$. a.u. represents arbitrary units. Other experimental parameters correspond to $K=0.1\Omega ,g=0.1\Omega ,\gamma =0.04\Omega$, and $\bar{\gamma }=0.1\Omega$

The schematic configuration of the experimental setup is illustrated in Fig. 3a (see detailed experimental setup in Fig. 6 of Materials and methods). Two fiber ring resonators A and B at the same length of $L=$11.6 m are coupled by a $2\times 2$ fiber coupler with a coupling ratio of $60:40$. In the absence of group velocity dispersion, each ring resonator supports a series of resonant frequencies ${\omega }_{n}^{a}={\omega }_{n}^{b}={\omega }_{n}={\omega }_{0}+n\Omega$. Here ${\omega }_{0}$ is a reference resonant frequency, $\Omega =2\pi {v}_{g}/L=2\pi \cdot 17.6\,{\rm{MHz}}$ is the free spectral range (FSR) with ${v}_{g}$ being the group velocity, $n=0,\pm 1,\pm 2$,… is the index of resonant frequency modes. Frequency modes in two rings at same frequency are coupled due to the fiber coupler with the coupling strength $K$. Different resonant frequency modes in each leg are coupled by the two electro-optic phase modulators (EOM1 and EOM2), with the phase modulation form ${W}_{A}(t)=g\cos (\Omega t+{\phi }_{a})$ and ${W}_{B}(t)=g\cos (\Omega t+{\phi }_{b})$, respectively. Here $g$ is the modulation amplitude, ${\phi }_{a},{\phi }_{b}$ are the modulation phases. In this architecture, resonant frequency modes ${\omega }_{n}^{a}$ and ${\omega }_{n}^{b}$ represent the lattice sites ${a}_{n}$ and ${b}_{n}$, and therefore a synthetic two-leg Hall ladder is constructed, with the effective magnetic flux $\phi ={\phi }_{b}-{\phi }_{a}$ in each plaquette (see Fig. 3b). In experiments, we can construct a passive non-Hermitian Hall ladder with different losses applied on two rings, (i.e., frequency modes on two legs). The resulting Hamiltonian of the non-Hermitian synthetic lattice in ${k}_{f}$-space is (refer to Supplementary Note 3)

7 $${H}_{{k}_{f}}=\left[\begin{array}{cc}-i{\gamma }_{a}+g\,\cos ({k}_{f}\Omega +{\phi }_{a}) & K\\ K & -i{\gamma }_{b}+g\,\cos ({k}_{f}\Omega +{\phi }_{b})\end{array}\right]$$

Here ${k}_{f}$ is the wave vector that is reciprocal to the frequency dimension, and thus it acts as the time variable^23^. As one round-trip time is equivalent to one Brillouin zone, the quasimomentum can be defined by $k={k}_{f}\Omega$^37^. ${\gamma }_{a}$ and ${\gamma }_{b}$ are the dissipations of the legs $a$ and $b$, respectively. In experiments, we tune the dissipation of leg $b$ to achieve ${\gamma}_{b}\,>\,{\gamma }_{a}$ by an additional electro-optic amplitude modulator without modulation in ring B, thus making this two-leg Hall ladder has on-site pseudo-gain and loss by taking ${\gamma }_{a}=-\gamma +\bar{\gamma }$ and ${\gamma }_{b}=\gamma +\bar{\gamma }$ with $\bar{\gamma }=({\gamma }_{a}+{\gamma }_{b})/2$ being the global loss. Thus, this synthetic lattice is equivalent to the theoretical model in Eq. (2) except that the global loss $\bar{\gamma }$. Since the non-Hermitian topological properties are not affected by the global loss^42^, our experimental configuration can be used to observe the antichiral currents discussed above.

To measure the currents in the frequency dimension, we inject a tunable continuous-wave laser from the input fiber of ring A (${s}_{{\rm{in}}}^{{\rm{A}}}$) to excite one mode ${\omega }_{0}^{a}$ (i.e., the 0-th site on the leg $a$) with a frequency detuning $\Delta \omega$ in the frequency dimension (see Fig. 3b). The currents on the legs $a$ and $b$ are governed by the ${J}_{a}$ and ${J}_{b}$ in Eq. (5), and finally reach the steady-state limit with the dissipation, which can be characterized by the steady-state mode distributions ${P}_{a}(n)$ and ${P}_{b}(n)$ on the legs $a$ and $b$, respectively. Nonzero currents in the two legs will result in asymmetric mode distributions about the central mode ${\omega }_{0}$ on each leg in the steady-state limit. To quantify the currents, we, therefore, define the steady-state currents as^36^

8 $${j}_{a(b)}=\sum _{n > 0}{P}_{a(b)}(n)-\sum _{n < 0}{P}_{a(b)}(n)$$

The nonzero current [${j}_{a(b)}\,\ne\,0$] reflects asymmetric mode distributions referring to the central mode ${\omega }_{0}$ on each leg caused by unidirectional currents in the frequency dimension^36^, and antichiral currents (co-propagating currents on two legs) can be demonstrated if the signs of steady-state currents on each leg are same, i.e., ${j}_{a},{j}_{b}\,>\, 0({j}_{a},{j}_{b} \,<\, 0)$, meaning the antichiral currents in the $+\hat{x}$ ($-\hat{x}$) direction. In experiments, we can obtain the steady-state mode distributions ${P}_{a}(n)$ and ${P}_{b}(n)$ by using the heterodyne detection method^36^. Specifically, we shift the frequency of the input laser by $\delta \omega =2\pi \cdot 200\,{\rm{MHz}}$ by an acousto-optic modulator and interfere it with the drop-port output fields of two rings (${s}_{{\rm{out}}}^{{\rm{A}}}$, ${s}_{{\rm{out}}}^{{\rm{B}}}$) respectively to obtain the interfering fields (see Fig. 6 in “Materials and methods” for details). The mode distributions ${P}_{a}(n)$ and ${P}_{b}(n)$ then can be obtained by a fast Fourier transform of the interfering fields, respectively. We scan the frequency of the input laser through the whole band structure to obtain ${P}_{a}(n)$ and ${P}_{b}(n)$ corresponding to energies of all bands. Figure 3c shows ${P}_{a}(n)$ and ${P}_{b}(n)$ for the non-zero effective magnetic flux of $\phi =0.5\pi$. We can see ${P}_{a}(n)$ and ${P}_{b}(n)$ are both biased to $n\,<\,0(n\,>\,0)$ for the input frequency detuning $\Delta \omega\,>\,0(\Delta \omega\,<\, 0)$, indicating the antichiral currents along $-\hat{x}(+\hat{x})$ or lower- (higher-) frequency regime. An example mode distributions near $\Delta \omega =-0.12\Omega$ are shown in Fig. 3d. One can see the distributions of steady-state modes on both rings give larger occupation in the higher frequency regime, indicating the positive antichiral currents. In Fig. 3e, we then plot the corresponding currents on the two legs (${j}_{a}$ and ${j}_{b}$) from Fig. 3c based on Eq. (8), where we observe positive antichiral currents $({j}_{a},{j}_{b} \,>\, 0)$ for the lower band $(\Delta \omega < 0)$ and negative antichiral currents $({j}_{a},{j}_{b} < 0)$ for the upper band $(\Delta \omega \,>\, 0)$, corresponding to the unidirectional frequency conversion in both rings, which are the hallmark of the antichiral currents in the synthetic non-Hermitian Hall ladder lattice. Such unidirectional frequency conversion can be robust against disorders (detailed discussion can be found in Supplementary Note 5), different from conventional conversion mechanisms^75,76^. As the currents are indirectly measured based on this passive system, the measured ${j}_{a},{j}_{b}$ are not exactly the same as the theoretical analysis in Fig. 2 (see Supplementary Note 4 for explanations). Note the amplitude of ${j}_{a}$ is larger than that of ${j}_{b}$, which is due to the initial excitation on the leg $a$. The signs of ${j}_{a}$ and ${j}_{b}$, however, are the same, which is the evidence of the antichiral currents. The existence of antichiral currents gives the key signature of the skin effect (see previous explanations in Fig. 2), resulting from the interplay between the effective magnetic flux and the on-site gain/loss. When the flux is zero, i.e., $\phi =0$, we observe ${j}_{a}={j}_{b}=0$ (see Fig. 3g), which shows no antichiral currents. Therefore, there is no skin effect in this case (antichiral currents for $\phi =\pi$ are also zero, seeing Supplementary Note 6).

We also obtain the band structures using the standard time-resolved band structure spectroscopy^28^. To obtain the band structures, we directly obtain the drop-port transmission spectrum from ring A $({s}_{{\rm{out}}}^{{\rm{A}}})$ after injecting the laser field in ring A. Then we break the transmission spectrum into different time slices with the time window $T=2\pi /\Omega$, which is the one round-trip time of ring A or B. By stacking up these time slices as a function of the frequency detuning, we obtain the transmission ${T}_{{\rm{out}}}^{{\rm{A}}}(\Delta \omega ,k)$, which is the band structure. Figures 3f and 3h show the measured band structures for $\phi =0.5\pi$ and $\phi =0$, respectively. The measured band structures exhibit the projection of whole band structure shown in Fig. 2a, d on the leg $a$ (see Supplementary Note 3). (Projection of band structure on leg $b$ can also be obtained if we inject the laser into ring B and get the drop-port transmission spectrum from the ring B.) The measured results show agreement with these of numerical simulations based on the actual passive system (refer to Supplementary Note 4).

**Fig. 4:**
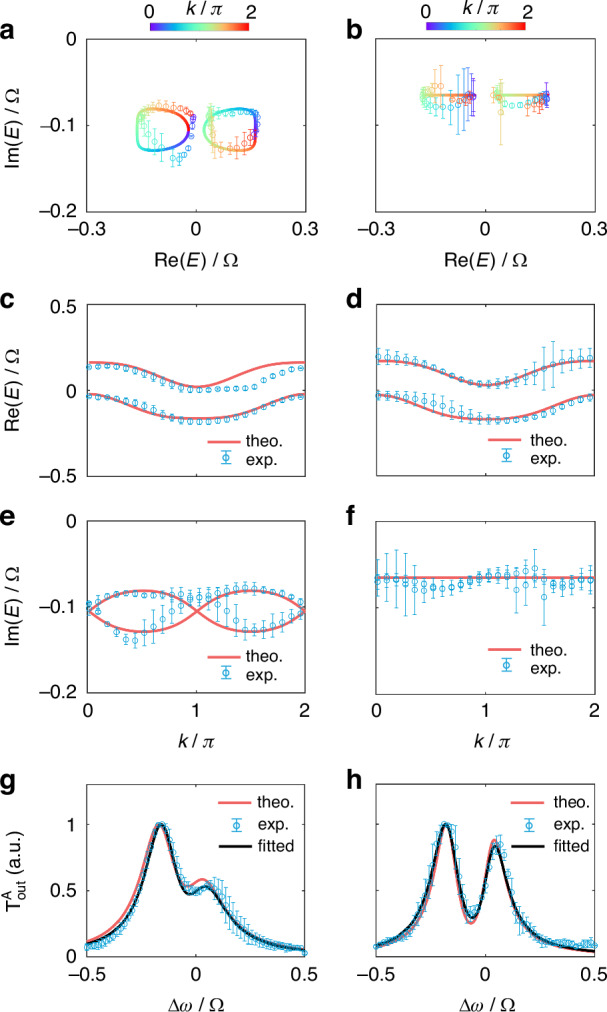
**Measurement of the topological windings. a**, **b** Measured and theoretical energy bands in the complex energy plane. Circles with error bars represent experimental results. The lines denote theoretical calculations. **c**, **d** Measured (blue circles with error bars) and theoretical (red lines) $\mathrm{Re}(E)$ and **e**, **f** ${\rm{Im}}(E)$. **g**, **h** Measured (blue circles with error bars), theoretical (red lines), and fitted transmission ${T}_{{\rm{out}}}^{{\rm{A}}}$ (black lines) at $k=\pi$. The left and right columns illustrate the non-Hermitian ($\gamma =0.04\Omega$) and Hermitian $(\gamma =0)$ cases with $\phi =0.5\pi$. Error bars are obtained from four independent measurements. Other parameters are same as those used in Fig. 3

Besides the antichiral currents, the topological winding of the energy bands can provide another evidence of the skin effect. For a fixed $k$, the transmission ${T}_{{\rm{out}}}^{{\rm{A}}}(\Delta \omega )$ exhibits a two-peak Lorentzian function of $\Delta \omega$ (see Fig. 3f, h), and has the form (refer to Supplementary Note 3)

9 $${T}_{{\rm{out}}}^{{\rm{A}}}(\Delta \omega )=\frac{{R}_{1}}{[{\rm{Re}}({E}_{1})-\Delta \omega {]}^{2}+[{\rm{Im}}({E}_{1}){]}^{2}}+\frac{{R}_{2}}{[{\rm{Re}}({E}_{2})-\Delta \omega {]}^{2}+[{\rm{Im}}({E}_{2}){]}^{2}}$$

where ${R}_{1}$ and ${R}_{2}$ are fitting constants. We can then obtain the real and imaginary parts of eigenenergies, $\text{Re}(E)$ and $\text{Im}(E)$, from the measured projected band structures by fitting the measured ${T}_{{\rm{out}}}^{{\rm{A}}}(\Delta \omega )$ with Eq. (9). Using this method, we extract the trace of $\mathrm{Re}(E)$ and ${\rm{Im}}(E)$ in the whole first Brillouin zone $k\in (0,2\pi ]$, and plot $\mathrm{Re}(E)$ and ${\rm{Im}}(E)$ in the complex energy plane to character the point-gap topology. In Fig. 4c, e, we showcase the experimentally obtained real $[\text{Re}(E)]$ and imaginary part $[\text{Im}(E)]$ of the band structures for $\phi =0.5\pi$, where the measured and fitted ${T}_{{\rm{out}}}^{{\rm{A}}}(\Delta \omega )$ as a function of $\Delta \omega$ at $k=\pi$ are shown in Fig. 4g. We plot the experimentally measured and theoretically simulated eigenenergies in the complex energy plane for the entire first Brillouin zone in Fig. 4a. One can see that $\text{Re}\left(E\right),\text{Im}(E)$ form two closed loops with the corresponding winding number $\pm 1$ from oppositely circulated trends of $[\text{Re}(E),\text{Im}(E)]$ versus $k$ from $0$ to $2\pi$, indicating the existence of the skin effect if an open boundary condition is applied to the synthetic lattice. The existence of the skin effect is the result of the interplay between the magnetic flux and the on-site gain/loss. As the comparison, for the Hermitian case with $\gamma =0$ and all other parameters being the same, we use the same method to obtain the $\text{Re}\left(E\right),\text{Im}(E)$ and plot results in Fig. 4 for the comparison. One can see that the trend of eigenenergy versus $k$ in the complex energy plane form lines (see Fig. 4b), presenting the absence of the skin effect.

**Fig. 5:**
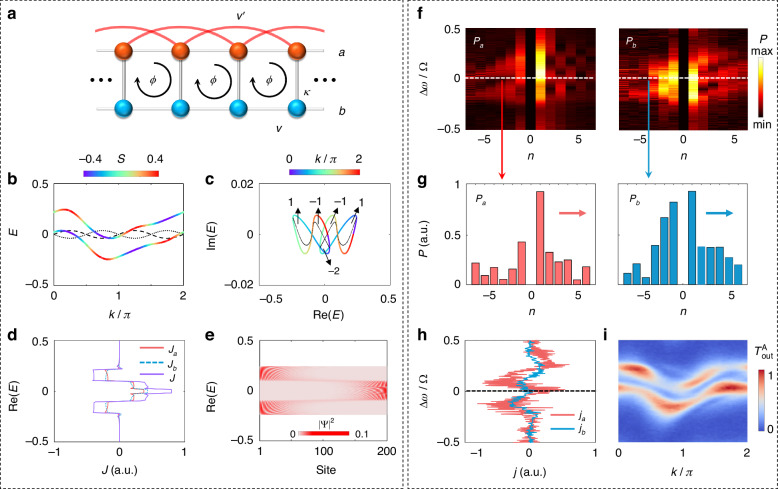
**Observation of antichiral currents with higher winding number. a** The Hall ladder lattice including next-nearest-neighbor coupling $v{\prime}$ on the leg $a$. **b** The corresponding band structure with real parts (solid lines) and imaginary parts (dashed line for the upper band and dotted line for the lower band). The imaginary parts have been enlarged by 5 times. **c** Energy spectra under the periodic boundary condition (colored line) and open boundary condition (black dots) where the numbers indicate the winding number $w$ in different loops. **d** The calculated currents ${J}_{a},{J}_{b}$ and $J={J}_{a}+{J}_{b}$. **e** Distributions of eigenstates for all the eigenenergies under the open boundary condition. The parameters in (**b**–**e**) are $\phi =0$, $v=0.06$, $\kappa =0.1$, $v{\prime} =0.04$, ${\phi }_{a}^{{\prime} }=-0.5\pi$, and $\gamma =0.02$. **f** Experimental measured steady-state frequency mode distributions ${P}_{a},{P}_{b}$ of two rings. **g** Measured ${P}_{a},{P}_{b}$ at the frequency detuning $\Delta \omega =0$. The red and blue arrows in the figure denote the directions of currents on each leg. **h,** **i** Experimentally measured steady-state currents ${j}_{a},{j}_{b}$ (**h**) and projected band structure (**i**). The experiments give parameters $K=0.1\Omega$, $g=0.12\Omega$, $g{\prime} =0.04\Omega$, $\bar{\gamma }=0.07\Omega$, and $\gamma =0.02\Omega$. a.u. arbitrary units

We can further introduce long-range couplings in the non-Hermitian Hall ladder lattice and observe antichiral currents with higher winding numbers. As an example, we consider adding next-nearest-neighbor (NNN) couplings with the coupling strength ${v}^{\prime}$ on the leg $a$, as shown in Fig. 5a. To experimentally achieve this lattice, we replace the phase modulation of EOM1 by ${W}_{A}(t)=g\cos (\Omega t+{\phi }_{a})+{g}^{\prime} \cos (2\Omega t+{\phi }_{a}^{{\prime} })$, with ${g}^{\prime}$ and ${\phi }_{a}^{{\prime} }$ being the NNN modulation amplitude and phase. We show the theoretical band structures, energy spectra under periodic and open boundary conditions, antichiral currents, and distribution of all the eigenstates under open boundary conditions in Fig. 5b–e. From Fig. 5c, we see the energy spectra under the periodic boundary condition form several loops, which are different from those under the open boundary condition, thus indicating the existence of the skin effect. We also show the calculated winding number $w$ in different loops in Fig. 5c, and see that higher winding number of $w=-2$ can exist near $\mathrm{Re}(E)=0$. Comparing Fig. 5d with Fig. 5e, we see that the direction of skin effect is consistent with the direction of antichiral currents. The amplitude of total currents $(J={J}_{a}+{J}_{b})$ according to $w=-2$ is twice as those for these eigenenergies with $w=\pm 1$ (see Fig. 5d). In experiments, we use the same experimental method to obtain the steady-state mode distributions on the two legs (${P}_{a},{P}_{b}$), as shown in Fig. 5f. The steady-state currents ${j}_{a},{j}_{b}$ then can be obtained using Eq. (8), as shown in Fig. 5h. An example of steady-state mode distributions ${P}_{a},{P}_{b}$ at $\Delta \omega =0$ (white lines in Fig. 5f) are shown in Fig. 5g. We see that ${P}_{a},{P}_{b}$ both have large occupations in higher-frequency modes, indicating the positive antichiral currents along $+\hat{x}$ direction (black dashed line in Fig. 5h). We also measure the projected band structure in Fig. 5i, which agrees with the theoretical result from tight-binding model analysis in Fig. 5b. These experimental results of steady-state mode distributions and antichiral currents match well with the numerical simulations (see Fig. S13 in the Supplementary Note 8). We also study other cases with NNN couplings in Supplementary Note 8. Our model may be further extended to higher dimensions by adding long-range couplings that are multiple of the FSR. In such cases, the synthetic frequency dimension can be folded to 2D/3D synthetic lattices^25,31,35^, which might bring opportunities for studying the higher-dimensional non-Hermitian physics with the skin effect in the optical regime.

## Discussion

Before the conclusion, we provide several side notes. The observed antichiral currents are also related to the persistent bulk currents^70^ in non-Hermitian systems. In particular, any one of the three phenomena, i.e., antichiral currents, skin effect, and the winding number, is the sufficient and necessary condition of the other two^70^. In other words, the non-zero antichiral currents related to each eigenenergy in our two-leg model demonstrate the existence of nonvanishing bulk currents, which can therefore predict the existence of the skin effect for a specific eigenenergy.

The mechanism leading to the observed non-Hermitian antichiral currents points out the possibility for realizing the skin effect, bipolar skin effect and hybrid second-order skin-topological effect in a gain-loss photonic system without asymmetric couplings^56,63–65^, which possesses its advantage in understanding the physics of the skin effect from the interplay between the effective magnetic flux and on-site gain/loss. Such mechanism though is demonstrated in the synthetic frequency lattice here, may also be achieved in other photonic platforms using different degrees of freedom of light^7,26,27^, or in different fields such as optomechanics^77^ and cold atoms^5^. Moreover, the non-Hermitian Hall ladder here takes the advantage of the tunability provided by synthetic dimensions to showcases the way for manipulating the skin effect and steering the unidirectional flow of light. Such flexibility may also be implemented in future studies of different non-Hermitian models with asymmetric couplings such as the Hatano-Nelson^56,57^ and non-Hermitian Su–Schrieffer–Heeger models^59–61^ using the synthetic frequency dimension. Although this frequency lattice in our system is infinite, we can create an open boundary condition using auxiliary rings^30^ to observe the skin effect (see Supplementary Note 7 for detailed discussion). The antichiral currents with edge currents in the same direction on both legs are fundamentally different from chiral currents and exist obviously in quasi-1D lattice (detailed discussion can be found in Supplementary Note 9), and might find potential applications in robust photonic devices with unidirectional frequency conversions and even selective amplifications at higher/lower frequency modes if average loss is tuned into gain.

To conclude, we have theoretically studied and experimentally observed non-Hermitian antichiral currents from the broken chirality^38^ in a tunable Hall ladder with the synthetic frequency dimension in the optical regime. Such antichiral currents, exhibiting the co-propagating feature on two legs, can be used to predict the corresponding non-Hermitian skin effect, which is also confirmed by measuring the topological windings of the energy bands. Note recent experimental works on the interplay between topology and non-Hermiticity in different systems^78,79^, our work operates in a synthetic space so light gets unidirectional frequency conversion in both rings. We further consider the addition of NNN couplings and study non-Hermitian antichiral currents therein. Our work therefore extends anomalous topological phenomena into non-Hermitian regime, which is different from Hermitian antichiral states in previous works^19–22^ and hence holds promise in further explorations of exotic high-dimensional non-Hermitian topology with the synthetic space.

## Materials and methods

### Experimental setup

**Fig. 6:**
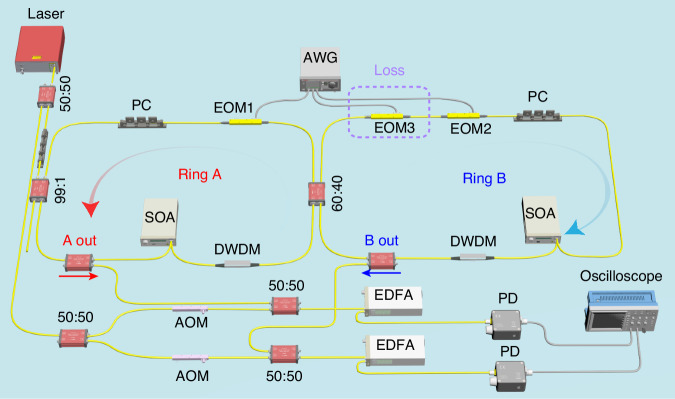
**Experimental setup**. Ring A and ring B are coupled by a $2\times 2$ fiber coupler. EOM1 and EOM2: electro-optic phase modulators. EOM3: electro-optic amplitude modulator, which is applied by a constant voltage to add an additional loss in the ring B. PC polarization controller, SOA semiconductor optical amplifier, AWG arbitrary waveform generator, EDFA erbium-doped optical fiber amplifier, PD photodiode, DWDM dense wavelength division multiplexing

The schematic of our experimental setup is shown in Fig. 6. A continuous-wave laser with a 200 kHz linewidth centered at 1550.92 nm is split to two parts by a $50:50$ fiber coupler. One part of the laser field is injected into ring A through a $2\,\times\,2$ fiber coupler with a coupling ratio $99:1$. The other part of the laser field is used to interfere with the drop-port output fields of the two fiber ring resonators for the mode distributions and band structure measurement. The laser frequency can be finely scanned over 5 GHz by applying a ramp signal on the frequency module. Two identical lithium niobate electro-optic phase modulators (EOMs) with 10 GHz bandwidth are driven by two arbitrary waveform generators (200 MHz bandwidth). The semiconductor optical amplifier (SOA) is used to compensate for the loss in each ring to obtain a high-quality factor. Two dense-wavelength division multiplexing (DWDM) band-pass filters (Channel 33, center wavelength 1550.92 nm) are utilized to effectively suppress the amplified spontaneous emission noise emanating from the SOA. The polarization controllers in both rings ensure that the polarization orientation of the laser in rings matches the principle axis of EOMs. An additional electro-optic amplitude modulator in the ring B adds an additional loss. Both fiber rings are coupled to through- and drop-ports to enable an independent calibration of the FSR of single ring. The two drop-port signals are directly sent to two fast InGaAs photodiodes ($850$ to 1650 nm with 10 GHz bandwidth) after optical amplification by an erbium-doped optical fiber amplifier (with a maximum gain of 12 dB) and then are sent to the oscilloscope (5 G samples/s with 1 GHz bandwidth) for measurements.

## Supplementary information

Supplementary information

## Data Availability

The supporting data for the findings in this study are available from the corresponding author upon reasonable request.
