# Supplementary material for: Observing non-Hermiticity induced chirality breaking in a synthetic Hall ladder
Source: Light Sci Appl. 2025 Jan 8;14:39. doi: 10.1038/s41377-024-01700-1 (PMC11707151; doi:10.1038/s41377-024-01700-1)
Supplement: Supplementary file 1 — Supplementary information [file 41377_2024_1700_MOESM1_ESM.pdf]

Supplementary Information for

Observing non-Hermiticity induced chirality breaking in a synthetic Hall ladder

Rui Ye[1*], Yanyan He[1*,†], Guangzhen Li[1*], Luojia Wang[1], Xiaoxiong Wu[1], Xin Qiao[1], Yuanlin Zheng[1], Liang Jin[2], Da-Wei Wang[3], Luqi Yuan[1‡], and Xianfeng Chen[1,4,5§]

[1]*State Key Laboratory of Advanced Optical Communication Systems and Networks, School of Physics and Astronomy, Shanghai Jiao Tong University, Shanghai, 200240, China*

[2]*School of Physics, Nankai University, Tianjin 300071, China*

[3]*Interdisciplinary Center for Quantum Information and Zhejiang Province Key Laboratory of Quantum Technology and Device, Department of Physics, Zhejiang University, Hangzhou 310027, China*

[4]*Shanghai Research Center for Quantum Sciences, Shanghai, 201315, China*

[5]*Collaborative Innovation Center of Light Manipulations and Applications, Shandong Normal University, Jinan, 250358, China*

These authors contributed equally: Rui Ye, Yanyan He, and Guangzhen Li

yanyhe@sjtu.edu.cn, yuanluqi@sjtu.edu.cn, xfchen@sjtu.edu.cn

1. Energy bands windings and antichiral currents for different γ

In this section, we discuss the energy bands windings in the complex plane and antichiral currents for different γ. We consider the flux $\phi = 0.5\pi$ and other parameters are the same as these in Fig. 1 of the main text. The energy band windings and total currents $J = J_a + J_b$ are shown in Fig. S1. We can see that the energy bands form a nonzero area and $J \neq 0$ as long as $\gamma \neq 0$, indicating that the antichiral currents emerge once $\gamma \neq 0$.

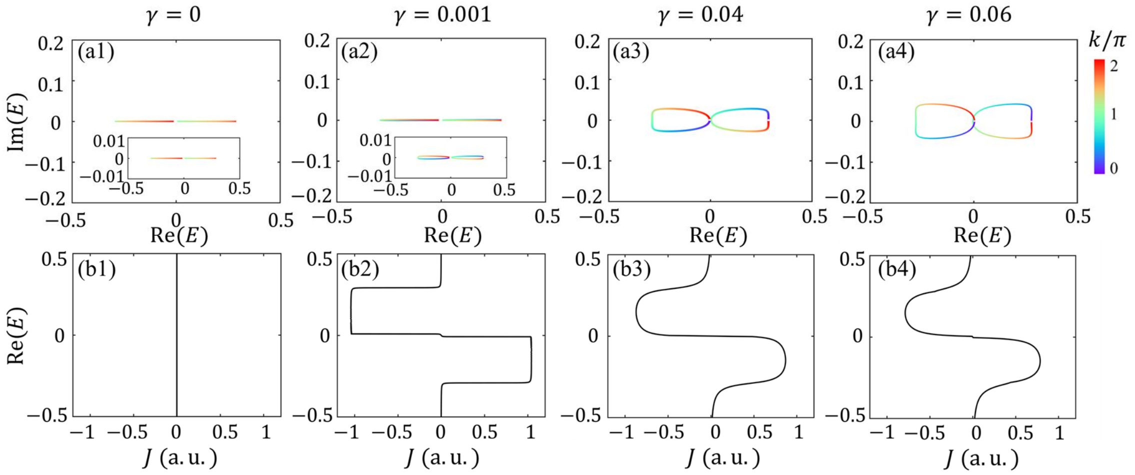

Fig. S1. The energy bands windings in the complex plane (a1-a4) and the corresponding total current $J = J_a + J_b$ (b1-b4) for different γ. Other parameters are the same as these in Fig. 1 of the main text.

Besides, from Fig. S1, one can see that the total currents are not necessarily quantized for the whole band energies when γ is large. The reason is that the currents are related to the density of states, which vary with γ. However, the total current J for a specific eigenenergy Re(E) is consistent with the winding number, thus is quantized. For example, when the winding number $w = 2$, the amplitude of J will be twice as these eigenenergy Re(E) with $w = 1$.

2. Theoretical energy band windings, currents, and eigenstates distribution for $\phi = 0.25\pi$ and $\phi = \pi$

We discuss the eigenstate distribution under open boundary condition and energy band windings under periodic boundary condition for $\phi = 0.25\pi$ and $\phi = \pi$, as shown in Fig. S2. Theoretical band structures, currents and distributions of eigenstates for all eigenenergies are shown in Fig. S3. The case of $\phi = 0.25\pi$ is similar with that of $\phi = 0.5\pi$ in the main text, except that for $\phi =$

0.25π, there is a range of eigenenergies near $\mathrm{Re}(E) = 0$ with $w = 0$, $J_a, J_b = 0$ [see Fig. S3(a2)], and thus the corresponding eigenstates are extended over the lattice sites (no skin effect), as shown in Fig. S3(a3). The case of $\phi = \pi$ is same as that of $\phi = 0$ in the main text, where the $J_a, J_b = 0$ for all the eigenenergies and thus there is no antichiral currents and skin effect [see Figs. S3(b2) and S3(b3)].

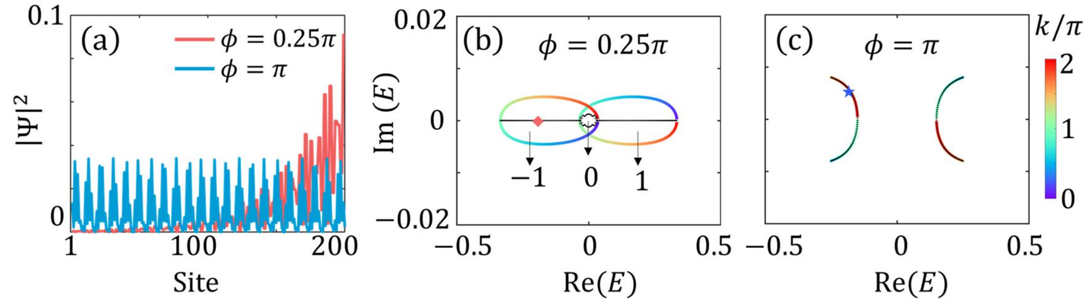

Fig. S2. (a) Eigenstates corresponding to one of eigenenergies with $\mathrm{Re}(E) = -0.2$ for different lattices under the open boundary condition with $\phi = 0.25\pi$ (red line) and $\phi = \pi$ (blue line). Energy spectra under the periodic boundary condition (colored lines) and open boundary condition (black dots) for lattices with $\phi = 0.25\pi$ (b) and $\phi = \pi$ (c), wherein the red diamond and blue stars denote one of the eigenenergies with $\mathrm{Re}\,(E) = -0.2$. The numbers in the figures indicate the value of w in different loops. Other parameters are the same as those in Fig. 1 of the main text.

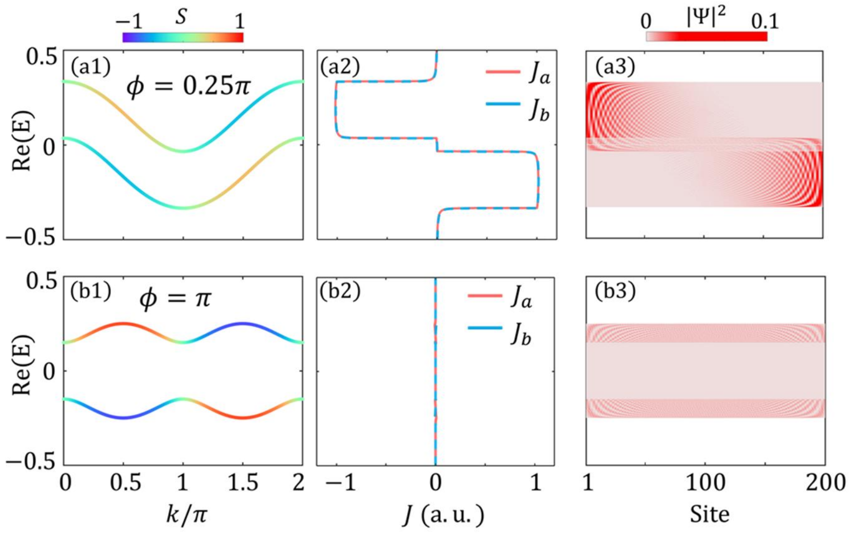

Fig. S3. Band structures (a1, b1). The color shows asymmetry ratio S. Currents $J_{a,b}$ (a2, b2). Distributions of the eigenstates for all eigenenergies under the open boundary condition (a3, b3). $\phi = 0.25\pi$ (a1-a3), $\phi = \pi$ (b1-b3). Other parameters are the same as those used for Fig. 2 in the main text.

3. Theory of projected band structure from time-resolved transmission

Following the method in previous studies [1, 2], we derive the projected band structures of this system. Considering the input field in ring A, the input-output coupled amplitude equations for amplitudes frequency modes in the systems are

$$\dot{a}_m = (-i\omega_m - \gamma_a)a_m - i\sum_n J^a_{n-m}(t)\, a_n - iKb_m + i\sqrt{\gamma_A} s^{\mathrm{A}}_{\mathrm{in}} e^{-i\omega t} \tag{S1}$$

$$\dot{b}_m = (-i\omega_m - \gamma_b)b_m - i\sum_n J^b_{n-m}(t)\, b_n - iKa_m \tag{S2}$$

$$\mathrm{s}^{\mathrm{A}}_{\mathrm{out}} = i\sqrt{\gamma_A}\sum_m a_m \tag{S3}$$

$$\mathrm{s}^{\mathrm{B}}_{\mathrm{out}} = i\sqrt{\gamma_B}\sum_m b_m \tag{S4}$$

where a_m (b_m) is the frequency mode amplitudes in ring A (B), $\gamma_A(\gamma_B)$ representes the coupling rates that all modes in ring A (B) couple to the input waveguide, and $\mathrm{s}^{\mathrm{A}}_{\mathrm{out}}$ $(\mathrm{s}^{\mathrm{B}}_{\mathrm{out}})$ represents the drop-port output amplitudes of the ring A (B), $J^{a(b)}_{n-m}(t) = J^{a(b)}_{n-m}(t+T)$ is the coupling strength induced by EOMs, with $T = 2\pi/\Omega$ being the period of the time modulation. We define a gauge transformation

$$a'_m = a_m \mathrm{e}^{i\omega_m t + i\omega t} = a_m e^{im\Omega t + i\omega t}, \qquad b'_m = b_m \mathrm{e}^{i\omega_m t + i\omega t} = b_m e^{im\Omega t + i\omega t} \tag{S5}$$

to express the coupled equations in a rotated basis as

$$\dot{a}'_m = -(i\omega + \gamma_a)a'_m - i\sum_{p+m} J^a_p(t)\, a'_{p+m} e^{-ip\Omega t} - iKb'_m + i\sqrt{\gamma_A} s^{\mathrm{A}}_{\mathrm{in}} e^{im\Omega t} \tag{S6}$$

$$\dot{b}'_m = -(i\omega + \gamma_b)b'_m - i\sum_{p+m} J^b_p(t)\, b'_{p+m} e^{-ip\Omega t} - iKa'_m \tag{S7}$$

where $p = n - m$. We then transform Eqs. (S6)-(S7) to k_f space by defining

$$a_{k_f} = \sum_m a'_m e^{-im\Omega k_f}, \qquad b_{k_f} = \sum_m b'_m e^{-im\Omega k_f} \tag{S8}$$

Then we have

$$\dot{a}_{k_f} = -(i\omega + \gamma_a)a_{k_f} - i\sum_p J^a_p(t)\, a_{k_f} e^{-ip\Omega t} e^{ip\Omega k_f} - iKb_{k_f} + i\sqrt{\gamma_A} s^{\mathrm{A}}_{\mathrm{in}} T\delta(t - k_f) \tag{S9}$$

$$\dot{b}_{k_f} = -(i\omega + \gamma_b)b_{k_f} - i\sum_p J^b_p(t)\, b_{k_f} e^{-ip\Omega t} e^{ip\Omega k_f} - iKa_{k_f} \tag{S10}$$

By defining the column vectors

$$\left|\mathbf{\Psi}_{k_f}\right\rangle = \begin{pmatrix} a_{k_f} \\ b_{k_f} \end{pmatrix}, \qquad |\mathbf{s}_{\rm in}\rangle = s_{\rm in}^{\rm A}\begin{pmatrix} 1 \\ 0 \end{pmatrix} \tag{S11}$$

we then can write the coupled equations as a more compact form as

$$i\left|\dot{\mathbf{\Psi}}_{k_f}\right\rangle = \omega\left|\mathbf{\Psi}_{k_f}\right\rangle + H_{k_f}\left|\mathbf{\Psi}_{k_f}\right\rangle - \sqrt{\gamma_A}T\delta\left(t-k_f\right)|\mathbf{s}_{\rm in}\rangle \tag{S12}$$

or

$$\left[\omega + \left(H_{k_f} - i\partial_t\right)\right]\left|\mathbf{\Psi}_{k_f}\right\rangle = \sqrt{\gamma_A}T\delta\left(t-k_f\right)|\mathbf{s}_{\rm in}\rangle \tag{S13}$$

where the k_f-space time-dependent Hamiltonian is

$$H_{k_f} = \sum_p \begin{bmatrix} -i\gamma_a + J_p^a(t)e^{-ip\Omega(t-k_f)} & K \\ K & -i\gamma_b + J_p^b(t)e^{-ip\Omega(t-k_f)} \end{bmatrix} \tag{S14}$$

Since the single frequency modulation, only $p = \pm 1$ terms remain. Under the rotating-wave approximation (RWA), we keep only the time-independent terms,

$$H_{k_f} = \begin{bmatrix} -i\gamma_a + g\cos\left(k_f\Omega + \phi_a\right) & K \\ K & -i\gamma_b + g\cos\left(k_f\Omega + \phi_b\right) \end{bmatrix} \tag{S15}$$

which is the Hamiltonian in Eq. (7) in the main text.

For a more general modulation without using the RWA, the Hamiltonian H_{k_f} is time-periodic. We define the Floquet eigenstates as

$$\left[-H_{k_f}(t) + i\partial_t\right]\left|\Psi_{k_f,n}(t)\right\rangle = \varepsilon_{k_f,n}\left|\Psi_{k_f,n}(t)\right\rangle \tag{S16}$$

where

$$\varepsilon_{k_f,n} = \varepsilon_{k_f} + n\Omega, \left|\Psi_{k_f,n}(t)\right\rangle = \begin{pmatrix} \psi_{k_f,n}^{\rm A} \\ \psi_{k_f,n}^{\rm B} \end{pmatrix} \tag{S17}$$

with $\left|\Psi_{k_f,n}(t)\right\rangle$ being a 2-component column vector that represents the pseudospin components of the Floquet eigenstate. We take the inner product with $\langle\Psi_{k_f,n}(t)|$ from the left on Eq. (S16). Note the inner product in the space of Floquet eigenstates is defined by

$$\langle\langle * \mid * \rangle\rangle_{\rm T} = \frac{1}{T}\int_0^T dt\langle * \mid * \rangle \tag{S18}$$

Then we have

$$\left\langle\left\langle \Psi_{k_f,n}(t)\middle|\left[\omega+\left(H_{k_f}-i\partial_t\right)\right]\middle|\mathbf{\Psi}_{k_f}\right\rangle\right\rangle_T =\frac{1}{T}\int_0^T dt\sqrt{\gamma_A}T\delta\left(t-k_f\right)\langle\Psi_{k_f,n}(t)|\mathbf{s}_{\text{in}}\rangle$$

$$=\sqrt{\gamma_A}\langle\Psi_{k_f,n}(t)|\mathbf{s}_{\text{in}}\rangle_{k_f=t} \tag{S19}$$

Then we obtain

$$\left\langle\left\langle \Psi_{k_f,n}(t)\middle|\mathbf{\Psi}_{k_f}\right\rangle\right\rangle_{\mathrm{T}} =\frac{\sqrt{\gamma_A}\left\langle\Psi_{k_f,n}(t)\middle|\mathbf{s}_{\text{in}}\right\rangle}{\omega-\varepsilon_{k_f,n}}|_{k_f=t} \tag{S20}$$

Since $\left|\mathbf{\Psi}_{k_f}\right\rangle$ is time-periodic, the Floquet eigenstates $|\Psi_{k_f,n}(t)\rangle$ form a complete basis for expanding them. Thus, we can finally write the output fields as

$$\mathrm{s}_{\text{out}}^{\mathrm{A}}e^{i\omega t}=i\sqrt{\gamma_A}\sum_m a'_m e^{-im\Omega t}=i\sqrt{\gamma_A}a_{k_f}$$

$$=i\sqrt{\gamma_A}(1,0)\times\left|\mathbf{\Psi}_{k_f}\right\rangle=i\sqrt{\gamma_A}(1,0)\times\sum_n\left|\Psi_{k_f,n}(t)\right\rangle\left\langle\left\langle\Psi_{k_f,n}(t)|\mathbf{\Psi}_{k_f}\right\rangle\right\rangle_T$$

$$=i\sqrt{\gamma_A}\sum_n\psi_{k_f,n}^{\mathrm{A}}(t)\frac{\sqrt{\gamma_A}\left\langle\Psi_{k_f,n}(t)\middle|\mathbf{s}_{\text{in}}\right\rangle}{\omega-\varepsilon_{k_f,n}}|_{k_f=t}. \tag{S21}$$

As ring A is excited, $|\mathbf{s}_{\text{in}}\rangle=s_{\text{in}}^{\mathrm{A}}(1,0)^T$. Hence, $\left\langle\Psi_{k_f,n}(t)\middle|\mathbf{s}_{\text{in}}\right\rangle=s_{\text{in}}^{\mathrm{A}}{\psi_{k_f,n}^{\mathrm{A}}}^*(t)$ and

$$\mathrm{s}_{\text{out}}^{\mathrm{A}}e^{i\omega t}=i\gamma_A\sum_n\psi_{k_f,n}^{\mathrm{A}}(t)\frac{s_{\text{in}}^{\mathrm{A}}{\psi_{k_f,n}^{\mathrm{A}}}^*(t)}{\omega-\varepsilon_{k_f,n}}|_{k_f=t}=i\gamma_A s_{\text{in}}^{\mathrm{A}}\sum_n\frac{\left|\psi_{k_f,n}^{\mathrm{A}}(t)\right|^2}{\omega-\varepsilon_{k_f,n}}|_{k_f=t} \tag{S22}$$

Similarly, we can obtain

$$\mathrm{s}_{\text{out}}^{\mathrm{B}}e^{i\omega t}=i\gamma_B s_{\text{in}}^{\mathrm{A}}\sum_n\frac{\psi_{k_f,n}^{\mathrm{B}}(t){\psi_{k_f,n}^{\mathrm{A}}}^*(t)}{\omega-\varepsilon_{k_f,n}}|_{k_f=t} \tag{S23}$$

The time-resolved normalized transmission is

$$T_{\text{out}}^{\mathrm{A}}(t)=\left|\frac{s_{\text{out}}^{\mathrm{A}}}{s_{\text{in}}^{\mathrm{A}}}\right|^2=\left|\gamma_A\sum_n\frac{\left|\psi_{k_f,n}^{\mathrm{A}}(t)\right|^2}{\omega-\varepsilon_{k_f,n}}|_{k_f=t}\right|^2=\sum_n\frac{\gamma_A^2\left|\psi_{k_f,n}^{\mathrm{A}}(t)\right|^4}{\left|\omega-\varepsilon_{k_f,n}\right|^2}|_{k_f=t} \tag{S24}$$

Under the conditions of $\gamma_a,\gamma_b\ll\Omega$ and $g\ll\Omega/2$, only the term that is closest to the input frequency contributed to the sum. Besides, we consider the frequency detuning $\Delta\omega=\omega-n\Omega$,

and the eigenvalues ε_{k_f} have two values, written as $\varepsilon_{k_f,i}$, with $i = 1,2$. Then, we can write the normalized time-resolved normalized transmission as

$$T_{\text{out}}^{\text{A}}(t) = \left|\frac{s_{\text{out}}^{\text{A}}}{s_{\text{in}}^{\text{A}}}\right|^2 = \sum_{i=1,2} \frac{\gamma_A^2 \left|\psi_{k_f,i}^{\text{A}}(t)\right|^4}{\left|\Delta\omega - \varepsilon_{k_f,i}\right|^2} |_{k_f=t} \quad \text{(S25)}$$

The simulated projected band structures are obtained from Eq. (S25), which indicates the projected band structure on the leg a that reads out from the ring A. The projected band structures on the leg b can also be obtained following the similar procedure by exciting the ring B.

Equation (S25) indicates that the transmission $T_{\text{out}}^{\text{A}}$ at a fixed time t show a two-peak Lorentzian function if we expand Eq. (S25) as

$$T_{\text{out}}^{\text{A}}(\Delta\omega) = \frac{\gamma_A \left|\psi_{k_f,1}^{\text{A}}(t)\right|^4}{\left[\text{Re}\left(\varepsilon_{k_f,1}\right) - \Delta\omega\right]^2 + \left[\text{Im}\left(\varepsilon_{k_f,1}\right)\right]^2} + \frac{\gamma_A \left|\psi_{k_f,2}^{\text{A}}(t)\right|^4}{\left[\text{Re}\left(\varepsilon_{k_f,2}\right) - \Delta\omega\right]^2 + \left[\text{Im}\left(\varepsilon_{k_f,2}\right)\right]^2} \quad \text{(S26)}$$

From Eq. (S26), we can write the fitting function of $T_{\text{out}}^{\text{A}}(\Delta\omega)$ as

$$T_{\text{out}}^{\text{A}}(\Delta\omega) = \frac{R_1}{[\text{Re}(E_1) - \Delta\omega]^2 + [\text{Im}(E_1)]^2} + \frac{R_2}{[\text{Re}(E_2) - \Delta\omega]^2 + [\text{Im}(E_2)]^2} \quad \text{(S27)}$$

The steady-state mode distributions $P_a(n), P_b(n)$ are obtained by taking the Fourier transform of Eqs. (S22) and (S23), respectively.

4. Numerical simulations of projected band structures and antichiral currents

In this section, we give the numerical simulations of the projected band structures and antichiral currents. The numerical simulations of the projected band structures on the leg a for different ϕ are shown in Figs. S4(a1)-S4(c1). The corresponding $P_a(n)$ and $P_b(n)$ are shown in Figs. S4(a2)-S4(c2) and Figs. S4(a3)-S4(c3), respectively. The corresponding steady-state currents are shown in Figs. S4(a4)-S4(c4). These simulation results agree well with the experimental results in Fig. 3 of the main text.

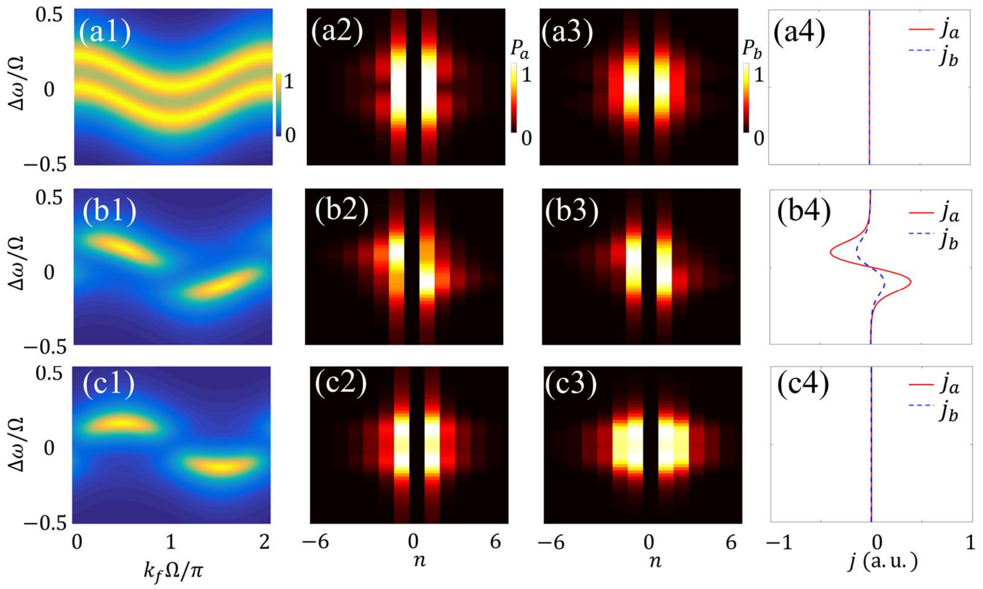

Fig. S4. Numerical simulations of projected band structures (a1-c1), steady-state frequency mode distributions $P_a(n)$ (a2-c2), $P_b(n)$ (a3-c3), and currents (a4-c4) for $\phi = 0$ (a1-a4), 0.5π (b1-b4), and π (c1-c4). The parameters are the same as those in Fig. 3 of the main text.

The experimental and simulation results of the antichiral currents are not the same as the these in Fig. 2 of the main text. We explain the main reasons below. In our experiment, we use a passive system to achieve the theoretical model and measure the steady-state currents. To measure the currents, we use the method in previous work of measuring the steady-state chiral currents in Hermitian synthetic frequency lattice [2]. When one frequency mode is pumped, the light will flow in the frequency dimension. Finally, the pump is balanced with the dissipation and the system reaches to a steady state. In such a steady-state limit, the frequency mode distribution will be asymmetric with the initial frequency mode due to the existing currents. We characterize the currents from the steady-state frequency mode distributions, based on Eq. (8) in the main text. This equation that characterizes the steady-state currents is an indirect method and not exactly same to the theoretical prediction of Eq. (5) in the main text. For the reasons above, the experimentally measured currents in Fig. 3 are not exactly the same as the theoretical analysis in Fig. 2 of the main text. To distinguish this difference, we have used J_a, J_b to represent the theoretical analysis and j_a, j_b to denote the experimental results in the main text. Nonetheless, the signature of the copropagating currents are demonstrated in the experiment.

5. Nonreciprocity in terms of the frequency conversion

The antichiral currents along the two-leg Hall ladder lattice characterize the nonreciprocity for the frequency conversion. To quantitatively describe the nonreciprocity in terms of frequency conversion, we introduce the isolation ratio $\mathrm{IR}_{a(b)} = 10\log_{10}[P^{+}_{a(b)}/P^{-}_{a(b)}]$ for the leg $a(b)$, where $P^{+}_{a(b)} = \sum_{n>0} P_{a(b)}(n)$ and $P^{-}_{a(b)} = \sum_{n<0} P_{a(b)}(n)$ indicate the total steady-state mode distributions at the higher and lower frequency. In Fig. S5, we show the experimental and theoretically expected isolation ratio on both legs for $\phi = 0.5\pi$. One can see that the maximum IR in terms of frequency conversion is near 5 dB.

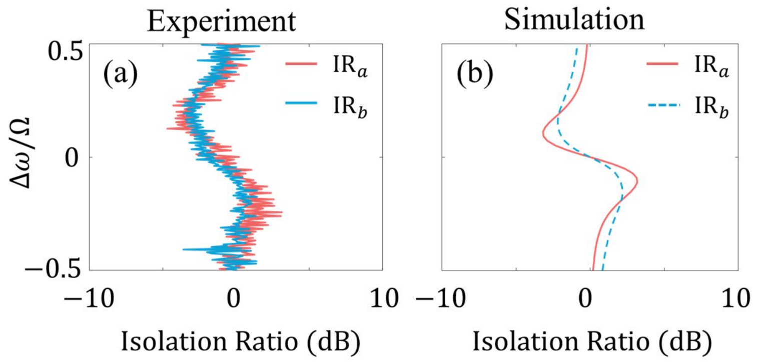

Fig. S5. (a) Experimentally measured isolation ratios IR_a and IR_b. (b) Expected isolation ratios IR_a and IR_b. Other parameters are the same as those in Fig. 3 of the main text.

We can also obtain the conversion efficiency from the steady-state frequency mode distributions. After the excitation of the input probe field on lattice site 0 (single frequency mode), the frequency modes are conversed to higher or lower frequency modes unidirectionally determined by the antichiral currents in the steady-state limit. We define the conversion efficiency $\eta_{a(b)}$ for the leg $a(b)$ by $\eta_{a(b)} = P^{\mathrm{con}}_{a(b)}/\sum_n P_{a(b)}(n)$, where $P^{\mathrm{con}}_{a(b)} = P^{-}_{a(b)}$ and $P^{\mathrm{con}}_{a(b)} = P^{+}_{a(b)}(n)$ for $\Delta\omega > 0$ and $\Delta\omega < 0$ respectively, representing the total normalized unidirectional converted frequency mode distributions corresponding to the input frequency mode. In Fig. S6, we show the theoretically calculated unidirectional conversion efficiency on both legs for $\phi = 0.5\pi$. One can see that the maximum conversion efficiency is near 40% with corresponding roughly 43% energy remains on the lattice site 0. The higher conversion efficiency can be achieved by decreasing the loss of the system due to the non-Hermitian nature here.

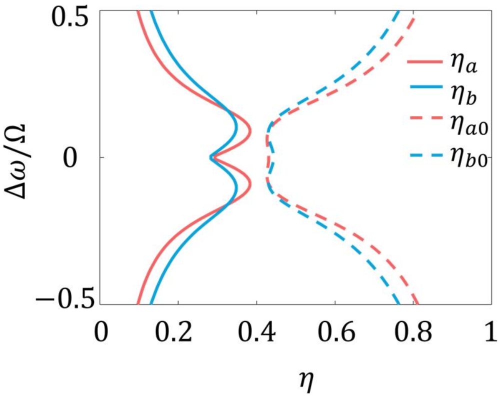

Fig. S6. Theoretically calculated unidirectional frequency conversion efficiency $\eta_a(\eta_b)$ on the leg $a(b)$ for $\phi = 0.5\pi$. $\eta_{a0}(\eta_{b0})$ denotes the energy left on the lattice site 0. Other parameters are the same as those in Fig. 3 of the main text.

Because of the intrinsic non-Hermitian topology nature in our system, we add analyses on the topological robustness of the non-Hermitian antichiral currents against disorders. Here, we provide additional numerical simulations of the antichiral currents under various disorders on the coupling strength, loss, and the on-site potentials to demonstrate the topological robustness. We replace the coupling strength K by $K(1 + r\delta - \delta/2)$, where r is a random value from 0 to 1 and δ characterizes the intensity of the disorder. In Fig. S7, we show the simulated steady-state antichiral currents and isolation ratios for different δ. One can see that the antichiral currents and isolation ratios remain almost unchanged, demonstrating robust unidirectional propagation of the antichiral currents that are against disorders on the coupling strength.

Following the above method, we consider the disorder on the loss γ_a by taking $\gamma_a = \gamma_a(1 + r\delta - \delta/2)$. The simulated steady-state antichiral currents and isolation ratios in terms of the frequency conversions are shown in Fig. S8. Similar to the case of the disorder on the coupling strength, the antichiral currents remain almost unchanged, indicating the antichiral currents that are robust against disorders on the loss.

Last, we discuss the disorder on the on-site potentials V_n of the leg a. We consider a random on-site potential $V_n = (r\delta - \delta/2)\Omega$ on the synthetic frequency lattice and simulate the steady-state antichiral currents and isolation ratios in Fig. S9. We can see that the antichiral currents are robust against disorders on the on-site potentials.

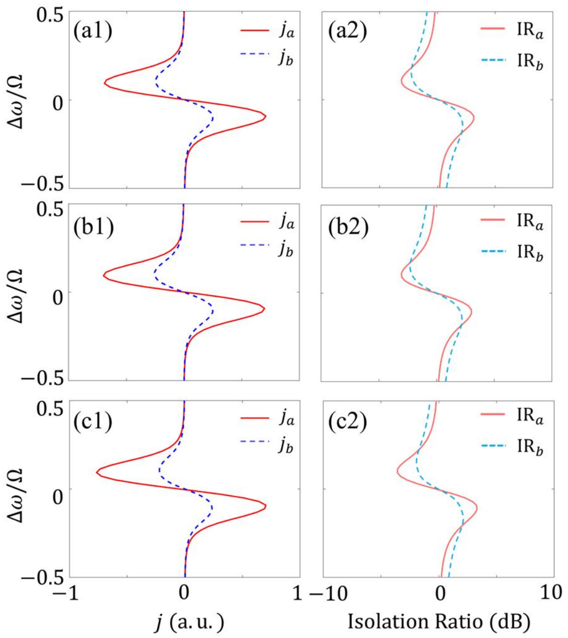

Fig. S7. Simulated antichiral currents (a1, b1, c1) and isolation ratio in terms of the frequency conversions (a2, b2, c2) for different disorder strength δ of the coupling strength $K(1 + r\delta - \delta/2)$, where $\delta = 0.1, 0.2, 0.4$ for (a1, a2), (b1, b2), (c1, c2), and r is a random value from 0 to 1. Other parameters are the same as those in Fig. 3 of the main text.

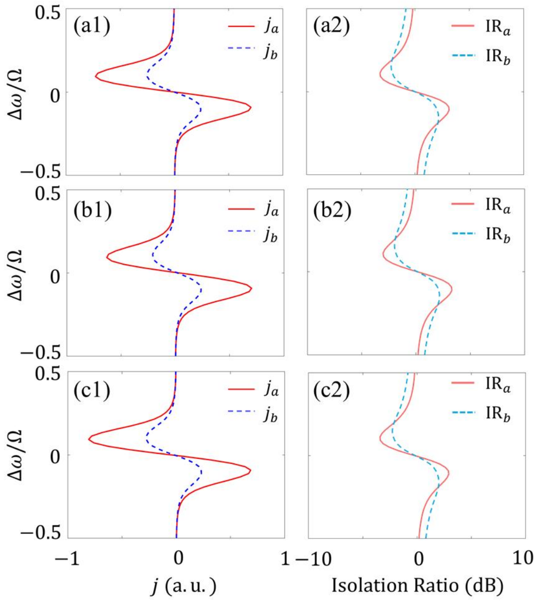

Fig. S8. Simulated antichiral currents (a1, b1, c1) and isolation ratio in terms of the frequency conversions (a2, b2, c2) for different disorder strength δ on the loss $\gamma_a(1 + r\delta - \delta/2)$, where $\delta = 0.1, 0.2, 0.4$ for (a1, a2), (b1, b2), (c1, c2), and r is a random value from 0 to 1. Other parameters are the same as those in Fig. 3 of the main text.

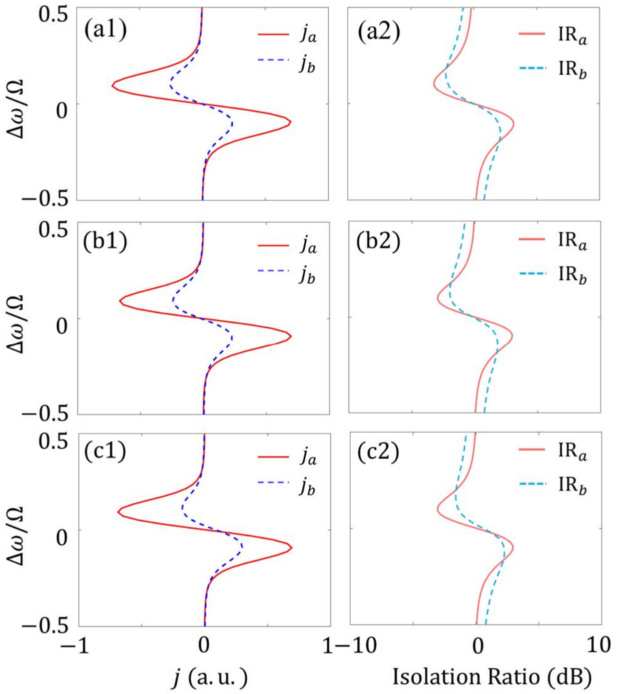

Fig. S9. Simulated antichiral currents (a1, b1, c1) and isolation ratios in terms of the frequency conversions (a2, b2, c2) for different disorder strength δ on the on-site potentials $V_n = (r\delta - \delta/2)\Omega$ of the leg a, where $\delta = 0.015, 0.03, 0.06$ for (a1, a2), (b1, b2), (c1, c2), and r is a random value from 0 to 1. Other parameters are the same as those in Fig. 3 of the main text.

6. Experimental band structures and currents for $\phi = \pi$

We add the additional measured band structures and currents for $\phi = \pi$ in Figs. S10(a) and S10(b), respectively. The experimental results agree well with the numerical simulations in Figs. S4(c1) and S4(c4), showing the absence of antichiral currents in this case.

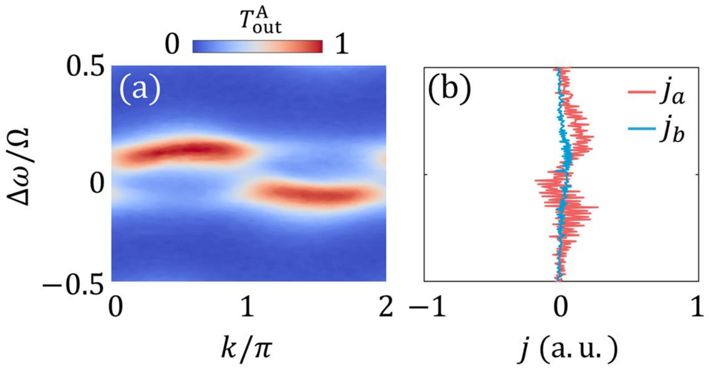

Fig. S10. Measured band structure (a) and currents (b) for $\phi = \pi$. Other experimental parameters are the same as those in Fig. 3 of the main text.

7. Proposal for creating boundaries and observing the skin effect

Although the Hall ladder lattice synthetized in the frequency dimension is infinite, we can create boundaries in this frequency lattice and observe the skin effect. To create an open boundary condition, we can add auxiliary rings in the main rings to create a boundary, which has been experimentally demonstrated in a recent work [3]. Based on this method, one can implement boundaries on the synthetic Hall ladder lattice by coupling two auxiliary ring resonators. As shown in Fig. S11(a), two auxiliary ring resonators C, D with identical length $L_{\mathrm{au}} = L/M$ are coupled to the ring resonators A and B, respectively. Here M is an integer. In this case, one can create a finite frequency-dimension Hall ladder lattice with the number of sites $2M$ [see Fig. S11(b)].

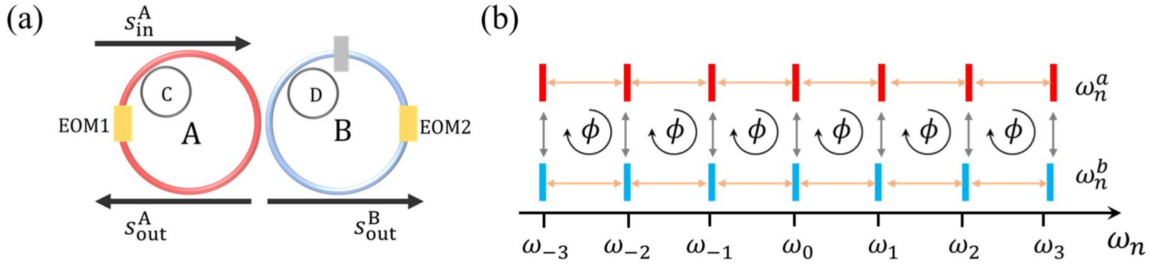

Fig. S11. (a) Configuration of creating boundaries on the synthetic Hall ladder lattice. Two auxiliary fiber ring resonators C and D are coupled with the ring A and B by two fiber couplers to create a finite frequency lattice in (b).

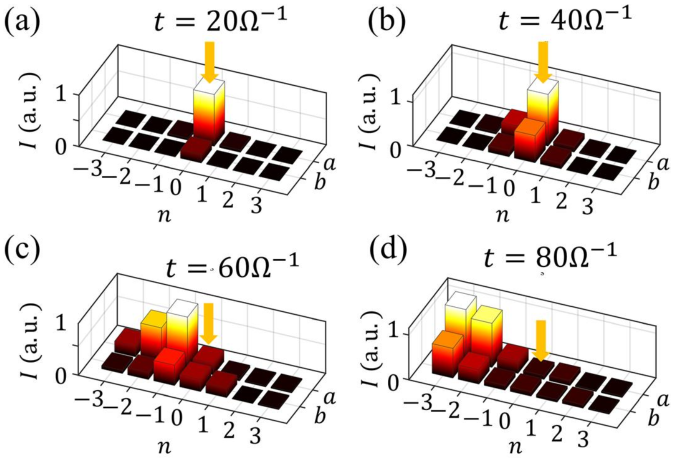

Fig. S12. Normalized mode distributions of a wavepacket on the synthetic Hall ladder lattice for different time $t = 20\Omega^{-1}$ (a), $t = 40\Omega^{-1}$ (b), $t = 60\Omega^{-1}$ (c), $t = 80\Omega^{-1}$ (d). The initial pulse has the parameters of $t_c = 35\Omega^{-1}, \sigma = 15\Omega^{-1}, \Delta\omega = 0.1\Omega$. The orange arrow denotes the initially excited frequency mode. The other parameters are the same as these in Fig. 3 (c) of the main text except $\bar{\gamma} = 0.04\Omega,\ \gamma = 0.03\Omega$.

After creating a finite frequency-dimension Hall ladder lattice, it is therefore possible to implement the non-Hermitian skin effect in this lattice. We can excite the frequency mode ω_0 using a Gaussian pulse $S_{\text{in}}(t) = e^{-(t-t_c)^2/\sigma^2 - i\Delta\omega t}$ and observe the mode distributions in this finite lattice. The non-Hermitian skin effect can be demonstrated by the signature of localized frequency modes near the boundary. In Fig. S12, we give the simulated results of the evolution of the Gaussian pulse.

We can see that the frequency modes on both legs all propagate to the lower frequencies (antichiral currents) and finally localize near the left boundary, thus demonstrating the non-Hermitian skin effect.

8. Antichiral currents and skin effect in the Hall ladder with next-nearest-neighbor couplings

We can further apply next-nearest-neighbor (NNN) couplings in the Hall ladder and study the antichiral currents and skin effect therein. The Hall ladder with NNN couplings can achieve antichiral currents with higher winding numbers, which paves the way for the further studies of the skin effect under higher winding numbers. As examples, we discuss NNN couplings on one leg and both legs here.

8.1 Only next-nearest-neighbor couplings on the leg a

We show the numerical results of projected band structures, steady-state distributions $P_a(n)$ and $P_b(n)$, and the steady-state currents in Fig. S13. It can be seen that the numerical simulations agree well with the experimental results in Fig. 5 of the main text.

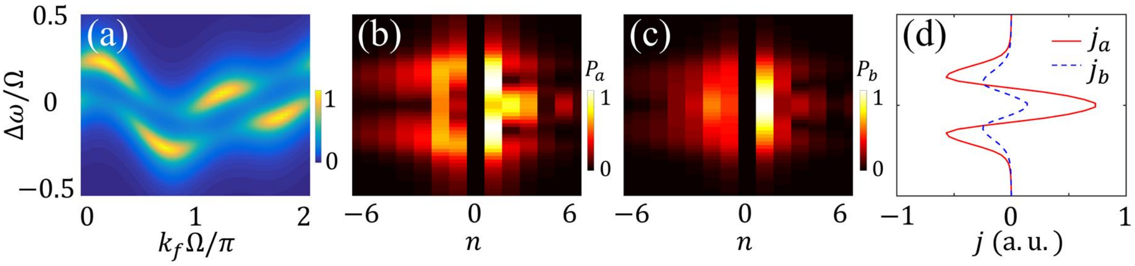

Fig. S13. Numerical simulations of projected band structures (a), steady-state frequency mode distributions $P_a(n)$ (b), $P_b(n)$ (c), and antichiral currents (d). The parameters are the same as those in Fig. 5(f) of the main text.

We next consider the case of nonzero effective magnetic flux $\phi = 0.5\pi$, the corresponding numerical simulations are shown in Fig. S14. We see that the antichiral currents still exist and are consistent with the skin effect. The total currents in this lattice is $J = J_a + J_b$, which determines the existence and directions of the skin effect.

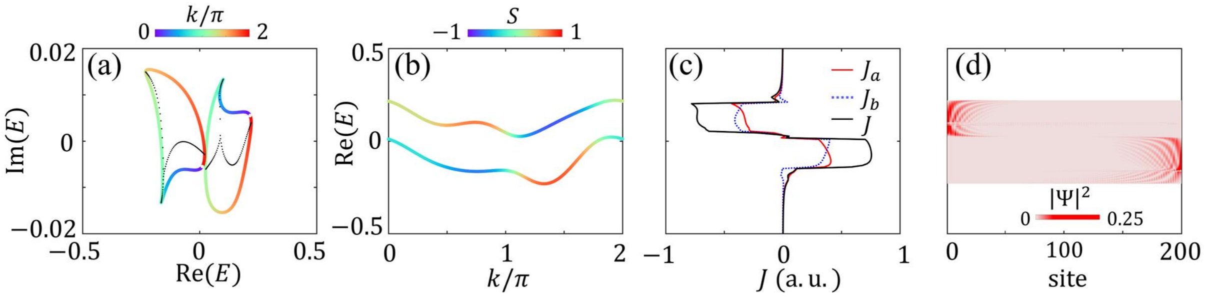

Fig. S14. (a) Energy spectra under the periodic boundary condition (colored line) and open boundary condition (black dots). (b) Band structures. (c) Currents J_a, J_b, $J = J_a + J_b$. (d) Distributions of the eigenstates for all eigenenergies under the open boundary condition. $\phi = 0.5\pi$, $\phi_a' = -0.25\pi$ and other parameters are same as those in Fig. 5(b) of the main text.

8.2 Next-nearest-neighbor couplings on both legs

Here we discuss the lattice formed by adding NNN couplings on both legs, as shown in Fig. S15. In this case, additional locally non-uniform effective magnetic flux $\pm\Theta$ is constructed in this lattice, which results in another effective magnetic flux Φ. There three effective magnetic fluxes ϕ, Θ, Φ satisfy the relationship $\Phi = 2(\Theta + \phi)$. We show the energy spectra, band structures, currents, and distributions of the eigenstates in Fig. S16. The numerical results demonstrate the existence of the antichiral currents in this lattice, and agree well with the skin effect. Note that this lattice can also achieve higher winding number of $w = -2$ [see Figs. S16(a1) and S16(a2)], the amplitude of total currents according to the $w = -2$ is twice as those for these eigenenergies with $w = \pm 1$ [see Figs. S16(c1) and S16(c2)], consistent with the previous analysis. Our synthetic frequency lattice thus exhibits great power in achieving lattices with long-range couplings, studying the dynamics under multiple effective magnetic fluxes.

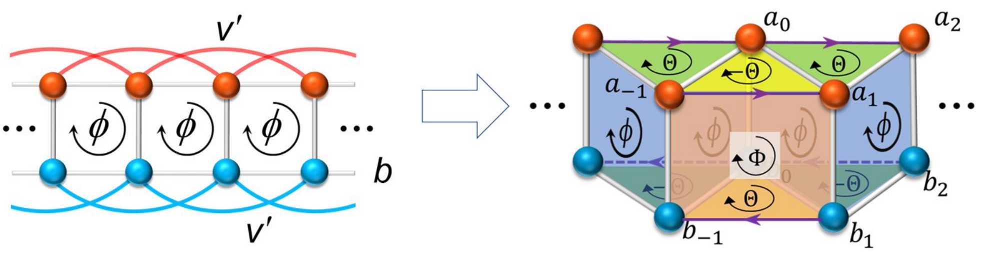

Fig. S15. The quasi-1D synthetic lattice with NNN couplings on both legs and the equivalent lattice with three effective magnetic fluxes ϕ, Θ, Φ.

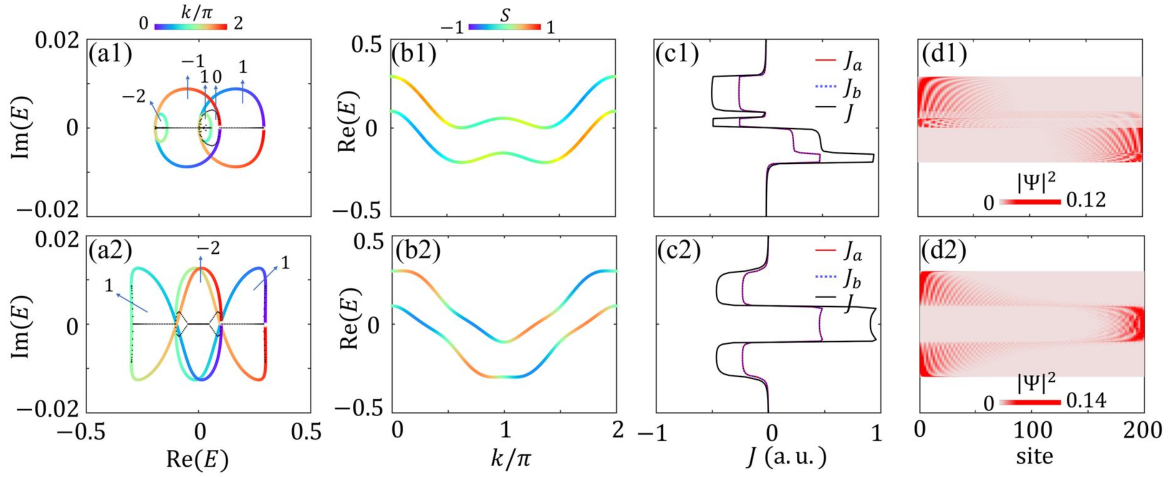

Fig. S16. (a1, a2) Energy spectra under the periodic boundary condition (colored line) and open boundary condition (black dots). (b1, b2) Band structures. (c1, c2) Currents J_a, J_b, $J = J_a + J_b$. (d1, d2) Distributions of the eigenstates for all eigenenergies under the open boundary condition. The parameters used in (a1-d1) are $\phi = 0.125\pi,\ \Theta = 0,\ \Phi = 0.25\pi,\ v = 0.06,\ \kappa = 0.1,\ v' = 0.04,\ \gamma = 0.02$. The parameters used in (a2-d2) are $\phi = 0,\ \Theta = \pi,\ \Phi = 0.5\pi,\ v = 0.1,\ \kappa = 0.1,\ v' = 0.04, \gamma = 0.02$.

9. Proposal for realizing two-dimensional non-Hermitian Hall lattice

In this section, we discuss the currents on two-dimensional (2D) Hall lattice and demonstrate that the antichiral currents can exist obviously in quasi-1D lattice. We can generalize this system into a 2D Hall lattice by extending the number of ring resonators. For example, we can achieve a 2D Hall lattice with N legs using N coupled-ring resonators, as shown in Fig. S17(a). The dissipations of the ring resonators can be tuned by adding additional electro-optic amplitude modulators to achieve the required staggered dissipation distributions, which is equivalent to the gain-loss lattice. Each ring resonators contain one phase electro-optic modulators with the modulation phase $\phi_l = l \cdot \phi$ with $l = 1, 2, \dots N$. In this case, a 2D Hall lattice with the effective

magnetic flux ϕ in each plaquette is constructed in the frequency dimension, as shown in Fig. S17(b).

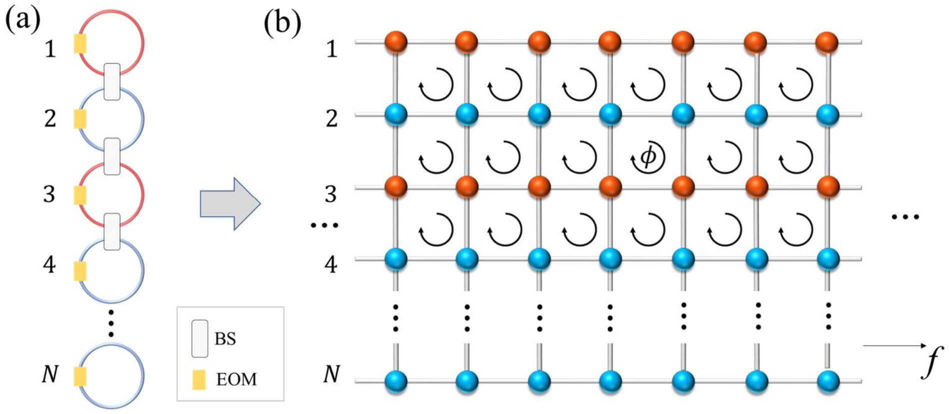

Fig. S17 (a) Theoretical configuration of realizing 2D non-Hermitian Hall ladder by coupling N ring resonators. (b)The synthetic 2D non-Hermitian Hall lattice with N legs. Red and blue balls denote the lattice sites with the on-site pseudo-gain and loss, respectively.

In a two-leg Hermitian Hall ladder without the gain/loss, two edge currents at the top and bottom edges propagate unidirectionally in opposite directions. Once the gain/loss is added and the lattice becomes a two-leg non-Hermitian Hall ladder in our model, the current on the bottom leg experiences loss while the current the top leg experiences gain and couple to the bottom leg, so the lattice exhibits the overall phenomena with antichiral currents. However, once the Hall ladder is generalized to 2D Hall lattice with the legs larger than 2, this antichiral currents may not exist due the existence of the bulk lattice which may prohibit the couplings between two edge currents.

To illustrate this point, we simulate the evolution of a Gaussian pulse on three different Hall lattices with the number of legs $N = 2, 4, 8$. The incident Gaussian pulse has the intensity distribution $S_{\text{in}}(t) = e^{-(t-t_c)^2/\sigma^2 - i\Delta\omega t}$. We excite the middle frequency site of the top and bottom leg respectively to see the directions of the currents on these two legs. The evolution of the pulse for the Hall lattices with $N = 2, 4, 8$ are shown in Figs. S18, S19, and S20, respectively. From Fig. S18, we can see that the antichiral currents exist in the two-leg Hall ladder ($N = 2$) due the couplings between these two legs. For the four-leg Hall lattice ($N = 4$), the antichiral currents still exist from the signatures of copropagating currents along the both legs (see Fig. S19). The reason is that the top leg with gain can still couple to the bottom with leg due to the few bulk lattice sites between top and bottom legs. However, when the bulk lattice becomes larger, for example, in the

eight-leg Hall lattice ($N = 8$), the currents on the top and bottom are difficult to couple each other, and thus the antichiral currents disappear. From Fig. S20, we can see that the current on the top with gain propagates to the lower frequencies, while the current on the bottom leg with loss propagate to the higher frequencies, thus indicating that the antichiral currents will not exist in this 2D Hall lattice.

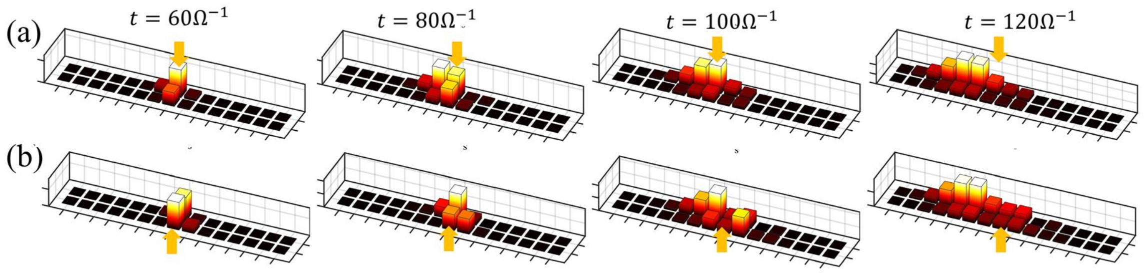

Fig. S18. The normalized intensity distribution of the pulse in the two-leg Hall lattice ($N = 2$) for the initial excitation on the top leg (a) and bottom leg (b). The initial pulse has the parameters of $t_c = 60\Omega^{-1}, \sigma = 20\Omega^{-1}, \Delta\omega = 0.1\Omega$. The orange arrows denote the initially excited frequency sites. Other parameters are the same as these in Fig. 3 of the main text except $\bar{\gamma} = 0.03\Omega$, $\gamma = 0.03\Omega$, and $\phi = 2\pi/3$.

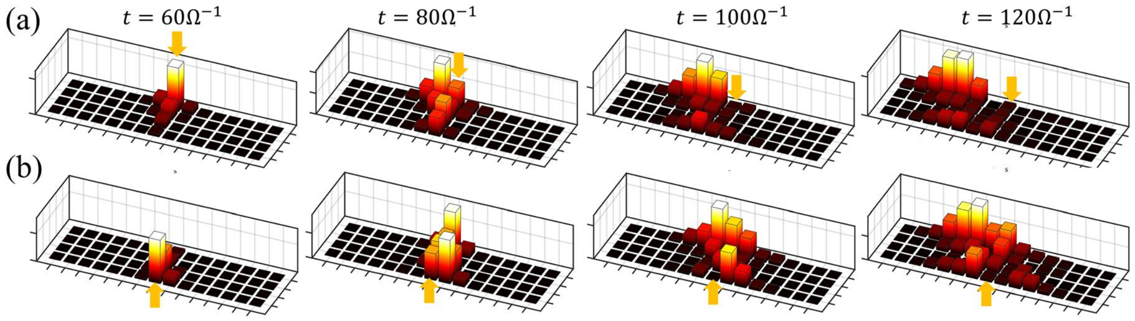

Fig. S19. The normalized intensity distribution of the pulse in the four-leg Hall lattice ($N = 4$) for the initial excitation on the top leg (a) and bottom leg (b). The orange arrows denote the initially excited frequency sites. Other parameters are the same as these in Fig. S18.

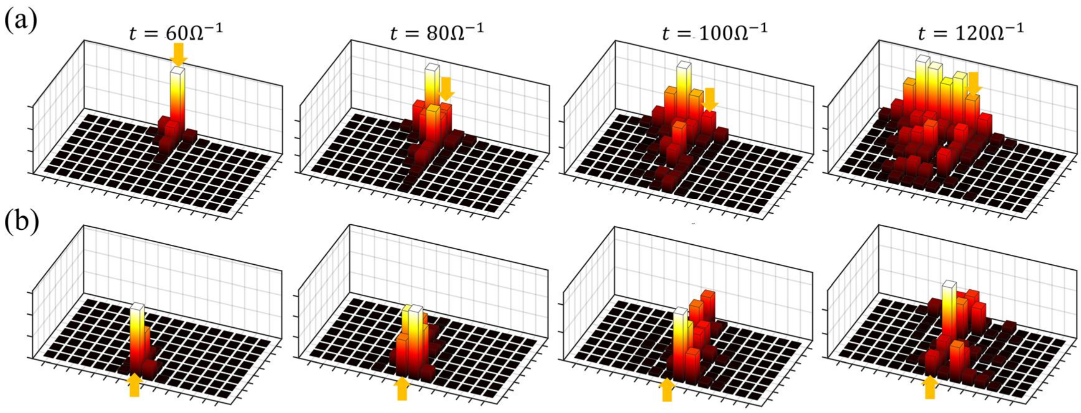

Fig. S20. The normalized intensity distribution of the pulse in the eight-leg Hall lattice ($N = 8$) for the initial excitation on the top leg (a) and bottom leg (b). The orange arrows denote the initially excited frequency sites. Other parameters are the same as these in Fig. S18.

References

[1] Dutt, A. et al. Experimental band structure spectroscopy along a synthetic dimension. *Nat. Commun.* **10**, 3122 (2019).

[2] Dutt, A. et al. A single photonic cavity with two independent physical synthetic dimensions. *Science* **367**, 59–64 (2020).

[3] Dutt, A. et al. Creating boundaries along a synthetic frequency dimension. *Nat. Commun.* **13**, 3377 (2022).
